# Reactivity of Dissolved Organic Matter with the Hydrated Electron: Implications for Treatment of Chemical Contaminants in Water with Advanced Reduction Processes

**DOI:** 10.1021/acs.est.3c00909

**Published:** 2023-05-04

**Authors:** Benjamin D. Fennell, Douglas Fowler, Stephen P. Mezyk, Garrett McKay

**Affiliations:** † Zachry Department of Civil & Environmental Engineering, Texas A&M University, College Station, Texas 77845, United States; ‡ Department of Chemistry and Biochemistry, California State University Long Beach, Long Beach, California 90840, United States

**Keywords:** dissolved organic matter, hydrated electron, kinetics, electron pulse radiolysis, reducing moieties

## Abstract

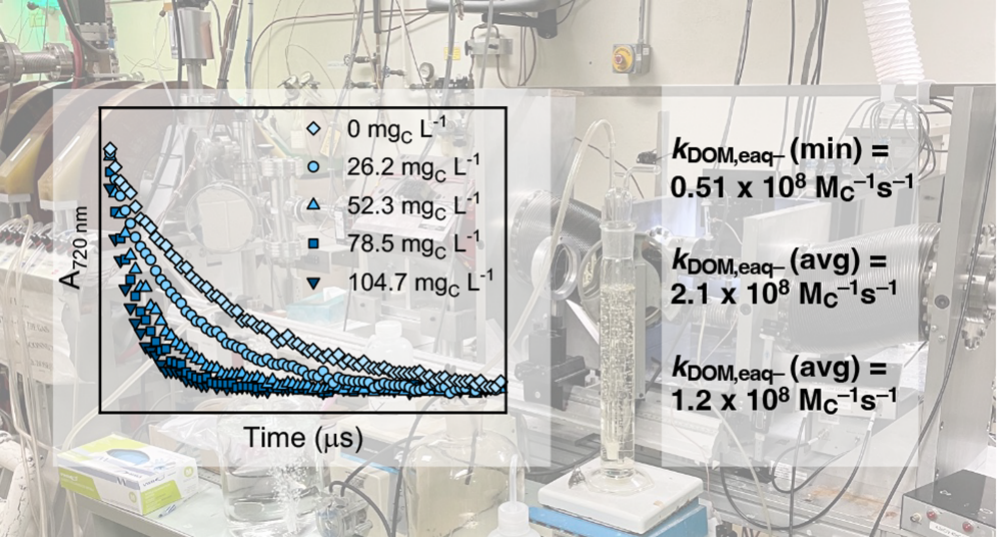

Advanced reduction processes (ARP) have garnered increasing attention for the treatment of recalcitrant chemical contaminants, most notably per- and polyfluoroalkyl substances (PFAS). However, the impact of dissolved organic matter (DOM) on the availability of the hydrated electron (e_aq_^–^), the key reactive species formed in ARP, is not completely understood. Using electron pulse radiolysis and transient absorption spectroscopy, we measured bimolecular reaction rates constant for e_aq_^–^ reaction with eight aquatic and terrestrial humic substance and natural organic matter isolates ( *k*_DOM,e_aq_^–^_), with the resulting values ranging from (0.51 ± 0.01) to (2.11 ± 0.04) × 10^8^ M_C_^–1^ s^–1^. *k*_DOM,e_aq_^–^_ measurements at varying temperature, pH, and ionic strength indicate that activation energies for diverse DOM isolates are ≈18 kJ mol^–1^ and that *k*_DOM,e_aq_^–^_ could be expected to vary by less than a factor of 1.5 between pH 5 and 9 or from an ionic strength of 0.02 to 0.12 M. *k*_DOM,e_aq_^–^_ exhibited a significant, positive correlation to % carbonyl carbon for the isolates studied, but relationships to other DOM physicochemical properties were surprisingly more scattered. A 24 h UV/sulfite experiment employing chloroacetate as an e_aq_^–^ probe revealed that continued e_aq_^–^ exposure abates DOM chromophores and e_aq_^–^ scavenging capacity over a several hour time scale. Overall, these results indicate that DOM is an important e_aq_^–^ scavenger that will reduce the rate of target contaminant degradation in ARP. These impacts are likely greater in waste streams like membrane concentrates, spent ion exchange resins, or regeneration brines that have elevated DOM concentrations.

## Abstract 2

Electron pulse radiolysis was used to measure the bimolecular reaction rate constant between the hydrated electron and eight dissolved organic matter isolates.

## 1 Introduction

Dissolved organic matter (DOM) is a complex, heterogeneous mixture of organic compounds naturally occurring in surface waters and groundwaters.^1^ DOM acts as a radical scavenger, thereby lowering the concentration of reactive species available for target contaminant degradation in advanced oxidation processes (AOP)^2,3^ and advanced reduction processes (ARP).^4−6^ Reactions between DOM and oxidizing radicals have been well characterized in the context of AOP, including hydroxyl radicals (^•^OH),^7,8^ sulfate radicals (SO_4_^•–^),^9^ carbonate radicals (CO_3_^•–^),^10^ chlorine radicals (Cl^•^ and Cl_2_^•–^),^11^ and bromine radicals (Br^•^ and Br_2_^•–^).^12^ In ultraviolet-advanced reduction processes (UV-ARP), the hydrated electron (e_aq_^–^) is considered to be the main reducing species^4,5,13−15^ with an aqueous reduction potential of −2.9 V.^16^ However, despite the growing interest in e_aq_^–^-mediated contaminant degradation, the reactivity of e_aq_^–^ with DOM is not well understood.

The scavenging of e_aq_^–^ by DOM represents an intrinsic limitation for the application of ARP in contaminated waters. ARP have been shown to degrade recalcitrant contaminants such as bromate,^15,17−22^ halogenated organic compounds,^4,13,14,23−31^ and per- and polyfluoroalkyl substances (PFAS),^32−42^ but the majority of these studies have been performed in relatively clean systems (e.g., lab-grade water). Some studies have tested ARP for treating concentrated waste streams produced from membrane filtration reject^42^ or regeneration of adsorbents,^43^ which have elevated DOM concentrations. Ren et al. also demonstrated that increasing the concentration of Aldrich humic acid inhibited the degradation of perfluorooctanoic acid in the UV/sulfite system.^6^ Another study conducted by Duan and Batchelor showed inhibited perchlorate degradation with increasing DOM concentration in the UV/sulfite ARP.^44^ As noted in these studies, and in parallel to the UV-AOP literature, this inhibition of target contaminant degradation can occur by DOM shielding the e_aq_^–^ sensitizer from absorbing UV photons, by scavenging e_aq_^–^, or by a combination of both processes. While the impact of light screening can be predicted based on absorbance measurements,^45^ accurate predictions of e_aq_^–^ scavenging by DOM are limited by the lack of reported bimolecular rate constants for this reaction.

The objectives of this study were to evaluate how the reactivity of e_aq_^–^ with DOM depends on DOM physicochemical properties, environmental conditions, and the prolonged e_aq_^–^ exposure typically encountered in ARP systems. These objectives were accomplished by quantifying bimolecular reaction rate constants (*k*_DOM,e_aq_^–^_) using electron pulse radiolysis for eight humic substance and natural organic matter (NOM) isolates in buffered solution at neutral pH and measuring *k*_DOM,e_aq_^–^_ values as a function of pH, ionic strength, and temperature for selected DOM samples. The isolates employed represent a wide range of physiochemical properties and chemical composition, being derived from both terrestrial and aquatic sources.^46^ Insights into the variability of *k*_DOM,e_aq_^–^_ among samples were gleaned by evaluating correlations to the physicochemical properties of DOM. Lastly, the impact of prolonged e_aq_^–^ exposure on DOM-e_aq_^–^ scavenging was evaluated in a 24 h UV/sulfite experiment conducted with Suwanee River natural organic matter II. Results from this study provide a means for estimating the e_aq_^–^ scavenging capacity of DOM in ARP, informing how this scavenging capacity changes with environmental conditions, and indicate that e_aq_^–^ scavenging by DOM can impact the efficacy of target contaminant degradation even after significant e_aq_^–^ exposure.

## 2 Materials and Methods

### 2.1 DOM Isolates, Chemicals, and Sample Preparation

Chemicals were purchased from Sigma-Aldrich or VWR and are listed in Table S1 in the Supporting Information (SI). In addition, eight humic substance and natural organic matter (NOM) isolates were purchased from the International Humic Substances Society (IHSS) and used for electron pulse radiolysis experiments, including Elliott Soil humic acid IV (ESHA IV), Pahokee Peat fulvic acid II (PPFA II), Pahokee Peat humic acid I (PPHA I), Pony Lake fulvic acid (PLFA), Suwannee River fulvic acid II (SRFA II), Suwannee River humic acid II (SRHA II), Suwannee River natural organic matter II (SRNOM II), and Upper Mississippi River natural organic matter (MRNOM). The IHSS catalog number for each isolate is available in SI Table S2. Additional IHSS catalog numbers were used for optical measurements (see SI Table S3) and *k*_DOM,e_aq_^–^_ comparison (SI Tables S5–S8), including Suwannee River fulvic acid III (SRFA III), Suwannee River humic acid III (SRHA III), and Elliott Soil humic acid V (ESHA V). All DOM stock solutions were prepared at a concentration of 200 mg L^–1^ in 10 mM dibasic phosphate that was adjusted to pH 5, 7, or 9 using HClO_4_, HCl, or NaOH. HClO_4_ was used for the pulse radiolysis studies. Ionic strength was varied using NaClO_4_. Ultrapure water (≥18.2 MΩ·cm) used for all experiments was obtained from either the Notre Dame University Radiation Laboratory reverse osmosis water treatment system or a Barnstead purification system (Thermo Fisher).

### 2.2 Analytical Measurements

Analytical measurements included pH, absorbance, dissolved organic carbon (DOC), and anion analysis. pH measurements were made with either an Orion Research pH/millivolt meter 811 (Notre Dame Radiation Laboratory) or a Thermo Scientific Orion Versa Star Pro combined with a micro Mettler Toledo LE422 pH probe (Texas A&M). A Cary-100 spectrophotometer (Agilent) with a 1 cm pathlength quartz cuvette was used to measure absorbance spectra, which were used to calculate specific ultraviolet absorbance at 254 nm (SUVA_254_) and spectral slope (*S*_300–600_) for the isolates. SI Text S1 provides additional measurement and calculation details for SUVA_254_ and *S*_300–600_. DOC measurements were performed by Hazen Huffman Laboratories in Golden, Colorado. Prior to DOC analysis, samples were acidified with trace-metal-grade nitric acid (70%) to pH ≤ 2.0 and stored at 4 °C. Anion analysis, except for sulfite, was conducted on a Dionex Integrion ion chromatography system equipped with a conductivity detector, a Dionex IonPac AS19 column (4 mm × 250 mm), a Dionex IonPac AG19 (4 mm × 50 mm) guard column, and a Dionex ADRS 600 (4 mm) suppressor. Anions were eluted with 20 mM KOH at a 1.0 mL min^–1^ flow rate and a 50 mA suppressor current. The column was temperature-controlled at 30 °C. Sulfite concentrations were quantified using the 5,5′-dithiobis(2-nitrobenzoic acid) assay and a thiol molar absorption coefficient of 14,000 M^–1^ cm^–1^, as described previously.^47,48^

### 2.3 Electron Pulse Radiolysis Techniques

*k*_DOM,e_aq_^–^_ values were measured using the linear accelerator system at the University of Notre Dame Radiation Laboratory.^49^ Numerous studies have utilized this approach to quantify bimolecular rate constants for reactions between organic compounds and various radical species.^50−54^ Methods previously established and utilized in this study for bimolecular reaction rate determination are briefly discussed below.

DOM stock solutions (200 mg L^–1^) were diluted with phosphate buffer in one of two dilution series (series 1: 160, 120, 80, and 40 mg L^–1^; series 2: 150, 100, and 50 mg L^–1^) in quartz cuvettes, purged with argon gas for at least 2 min, and sealed with glass stoppers. Water radiolysis via 7 ns electron pulses yielded 0.27 μmol e_aq_^–^ per J of energy absorbed.^16^ The transient e_aq_^–^ decays were monitored at 720 nm on a microsecond time scale for the dilution series as well as a phosphate buffer blank (DOM at 0 mg L^–1^) also purged with argon. Transient decay traces were averaged (∼30 traces) and fit with a first-order exponential decay plus baseline model to extract the pseudo-first-order decay constants of e_aq_^–^ (*k*′), which were then plotted vs the DOM concentration to obtain *k*_DOM,e_aq_^–^_. In this analysis, the change in *k*′ is due solely to the change in DOM concentration because the scavenging capacity of the background solvent is constant. Bimolecular rate constants were normalized to carbon concentration using the carbon mass % provided by the IHSS.^55^ Additional details involving pulse radiolysis techniques are discussed in SI Text S2.

### 2.4 Photochemical Irradiation Experiments

Irradiation experiments were conducted in duplicate immersion well reactors (Ace Glass) with an exterior glass body and interior quartz sleeve. Reactors were filled with ultrapure water (∼590 mL) and 1.0 mM borate buffer (pH 10.0) and purged with nitrogen gas for at least 45 min prior to and during experiments. The temperature in the reactors was controlled at 20 °C using a recirculating chiller. A low-pressure Hg, non-ozone forming lamp (10 W LSE Lighting GPH212T5L/4P) was powered on for at least 15 min and briefly turned off before concentrated stock solutions of sulfite, SRNOM II, and monochloroacetic acid (MCAA) were added to the reactor. UV/sulfite experiments were performed at a pH at least 2 pH units above the p*K*_a_ of HSO_3_^–^ (p*K*_a_ = 7.2) and under anerobic conditions to minimize e_aq_^–^ scavenging impacts of HSO_3_^–^, H^+^, and O_2_.^16,45,56,57^ After spiking, the solution was mixed for at least 30 s and stirring was maintained throughout experiments using a magnetic stir bar at 400 rpm. Experiments were initiated by turning on the lamps and collecting aliquots of solution using a stainless-steel needle and syringe. Samples were collected in either falcon tubes or 1.5 mL polypropylene vials and stored at 4 °C before analysis. UV irradiance was measured as 1.26 × 10^–8^ Es cm^–2^ s^–1^ using uridine actinometry^58^ and the previously measured average reactor pathlength was determined as 2.23 cm using the H_2_O_2_ method.^47,59,60^

## 3 Results and Discussion

### 3.1 DOM Isolate and e_aq_^–^ Bimolecular Rate Constant Measurements

**Figure 1:**
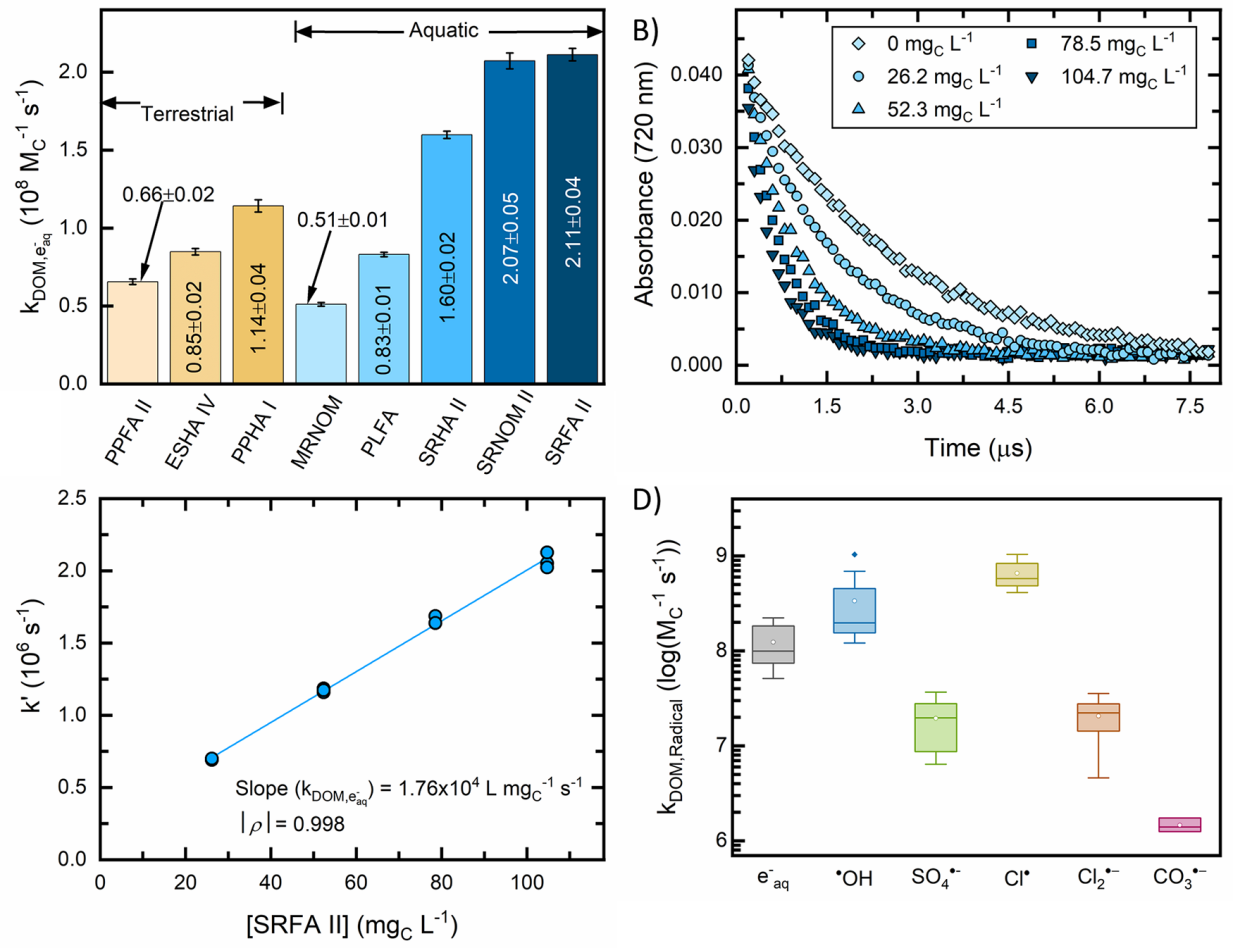
Bimolecular rate constant measurements between e_aq_^–^ and DOM isolates (*k*_DOM,e_aq_^–^_). Isolates include Elliott Soil IV humic acid (ESHA IV), Pahokee Peat II fulvic acid (PPFA II), Pahokee Peat I humic acid (PPHA I), Upper Mississippi River natural organic matter (MRNOM), Pony Lake fulvic acid (PLFA), Suwannee River II humic acid (SRHA II), Suwannee River II natural organic matter (SRNOM II), and Suwannee River II fulvic acid (SRFA II). *k*_DOM,e_aq_^–^_ in (A) were determined by measuring (B) transient absorption decay kinetics of e_aq_^–^ at 720 nm for various [DOM] and plotting (C) pseudo-first-order rate constant as a function of [DOM]. (B, C) Data for SRFA II only. The solid line in (C) represents a linear fit to the data using the least squares method with the slope reported as the *k*_DOM,e_aq_^–^_. Similar (C) plots for other DOM isolates are found in SI Figure S1. Error bars in (A) represent the standard error of the slope in (C). *k*_DOM,e_aq_^–^_ were compared to bimolecular rate constants between other radicals^7−11^ and DOM isolates in (D). DOM-e_aq_^–^ experiments conducted at pH 7.0 ± 0.1, 22 ± 2 °C, and 10.0 mM phosphate buffer. All other radical experiments in (D) were conducted at pH 7.0, room temperature, and varying concentrations of phosphate buffer.

*k*_DOM,e_aq_^–^_ values at pH 7.0 and 22 ± 2 °C varied by approximately a factor of 4, ranging from (0.51 ± 0.01) to (2.11 ± 0.04) × 10^8^ M_C_^–1^ s^–1^ (Figure 1A). *k*_DOM,e_aq_^–^_ values were determined by finding the pseudo-first-order rate constant from the transient e_aq_^–^ decay data (Figure 1B) and plotting the pseudo-first-order rate constant against the DOM concentration (Figure 1C). A linear fit to the data in Figure 1C yields *k*_DOM,e_aq_^–^_ as the slope with the *y*-intercept representing any e_aq_^–^ scavengers other than DOM (e.g., H^+^ in acidic conditions) present in the background solvent (see SI Text S2 for additional discussion). SI Figure S1 contains similar pseudo-first-order plots for the other DOM isolates and SI Text S3 discusses the minimal impact of IHSS catalog number on DOM-specific *k*_DOM,e_aq_^–^_ values.

*k*_DOM,e_aq_^–^_ values for soil humic substance isolates ranged from (0.66 ± 0.02) × 10^8^ M_C_^–1^ s^–1^ (PPFA II) to (1.14 ± 0.04) × 10^8^ M_C_^–1^ s^–1^ (PPHA I) and largely overlap with those of aquatic isolates, which ranged from (0.51 ± 0.01) × 10^8^ M_C_^–1^ s^–1^ (MRNOM) to (2.11 ± 0.04) × 10^8^ M_C_^–1^ s^–1^ (SRFA II). Soil humic acid *k*_DOM,e_aq_^–^_ values were lower than that for SRHA II, an aquatic humic acid. The *k*_DOM,e_aq_^–^_ value for PPFA II, a terrestrial fulvic acid, was also lower than the *k*_DOM,e_aq_^–^_ for SRFA II, an aquatic fulvic acid. However, not all *k*_DOM,e_aq_^–^_ values for aquatic isolates were higher than isolate terrestrial *k*_DOM,e_aq_^–^_ values. For example, MRNOM and PFLA (aquatic origin) had lower *k*_DOM,e_aq_^–^_ values than PPHA I (soil origin). Isolates from the Suwannee River had the largest *k*_DOM,e_aq_^–^_ values, with SRFA II and SRNOM II exhibiting similar reactivity and SRHA II being ∼20% lower. Overall, while *k*_DOM,e_aq_^–^_ is variable among these DOM samples, there is no clear trend between isolation procedure (humic substance *vs* natural organic matter) or source (aquatic *vs* soil).

The range of *k*_DOM,e_aq_^–^_ values reported on an M_C_^–1^ s^–1^ basis falls within the range of bimolecular reaction rate constants reported in the literature for oxidizing radicals’ reaction with DOM (Figure 1D and SI Table S4).^7−11^ On average, *k*_DOM,e_aq_^–^_ values were exceeded only by ^•^OH and Cl^•^ values. DOM is a primary sink for oxidizing radicals in sunlit waters and advanced oxidation processes. In anaerobic systems, such as electron transfer in anaerobic bottom waters and sediments or engineered systems like ARP, DOM will be an important e_aq_^–^ scavenger. Based on an average of the values measured for humic substance and NOM isolates, we recommend a *k*_DOM,e_aq_^–^_ value of 1.2 × 10^8^ M_C_^–1^s^–1^ (1.0 × 10^4^ L mg_C_^–1^ s^–1^). Employing this value yields an e_aq_^–^ scavenging capacity of 1.0 × 10^5^ s^–1^ at a dissolved organic carbon concentration of 10 mg_C_ L^–1^.

### 3.2 Impact of pH, Temperature, and Ionic Strength on *k*_DOM,e_aq_^**–**^_

**Figure 2:**
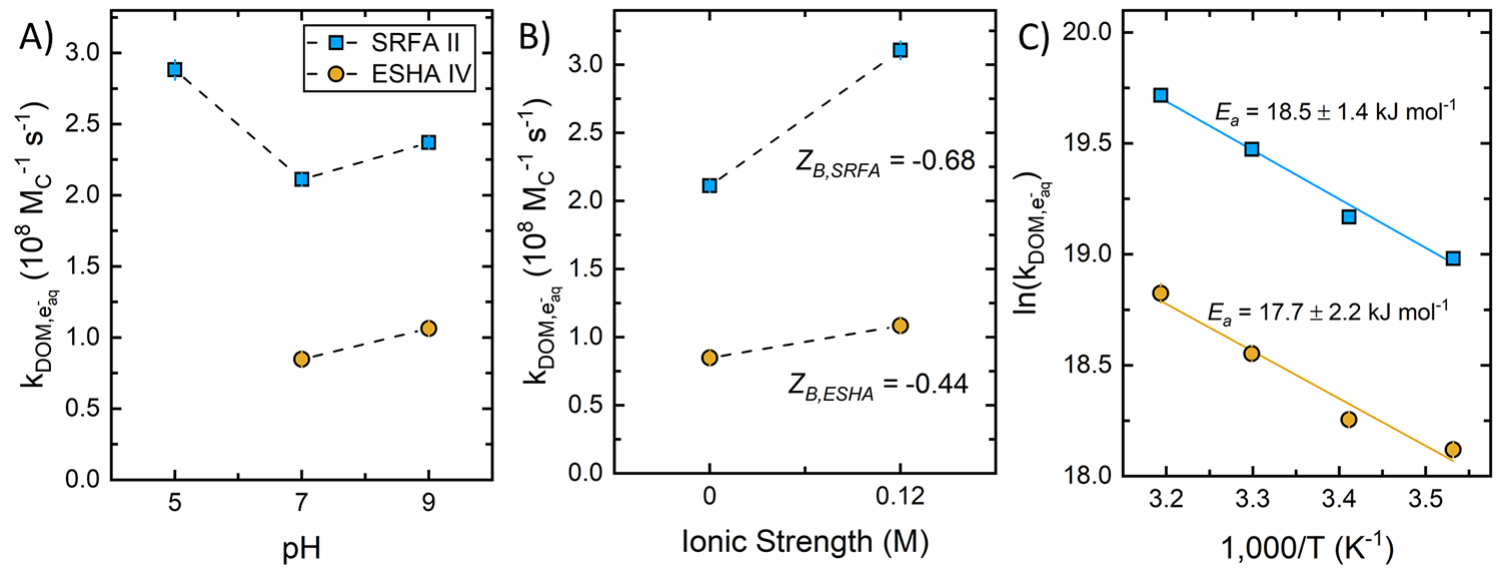
Influence of (A) pH, (B) ionic strength, and (C) temperature on bimolecular rate constants for SRFA II and ESHA IV. Experiments conducted at 22 ± 2 °C, pH 7.0 ± 0.1, and 10.0 mM dibasic phosphate buffer unless otherwise specified. The *Z*_B_ in (B) was calculated from the Brønsted-Bjerrum equation (eq 3.1) using the charge of e_aq_^–^ (i.e., *Z*_A_ = −1). Error bars represent the standard error of the bimolecular rate constant (majority of error bars are within markers). Additional plots of the pseudo-first-order rate constant against the DOM concentration for each pH, temperature, and ionic strength condition are provided in SI Figures S3 and S4.

We evaluated the impact of pH, temperature, and ionic strength on *k*_DOM,e_aq_^–^_ for two isolates, SRFA II and ESHA IV (Figure 2). Increasing pH from 7 and 9 caused small but significant increases in *k*_DOM,e_aq_^–^_ for both ESHA IV and SRFA II (Figure 2A, between 1.1- and 1.3-fold). Conversely, increasing pH from 5 and 7 caused a 1.4-fold decrease in *k*_DOM,e_aq_^–^_ for SRFA II.

The impact of pH on *k*_DOM,e_aq_^–^_ may be attributed to changes in the reactivity of DOM moieties at different protonation states, the impact of ionization on DOM molecular size, and the accessibility of reducible moieties to e_aq_^–^. Protonation of carboxylic acids generally increases the e_aq_^–^ bimolecular rate constant (e.g., acetic acid, *k* = 2 × 10^8^ M^–1^ s^–1^; acetate, *k* < 1 × 10^6^ M^–1^ s^–1^).^16^ This could explain the decrease in *k*_DOM,e_aq_^–^_ for SRFA II between pH 5 and 7 (we were unable to measure *k*_DOM,e_aq_^–^_ at pH 5 for ESHA IV). Increasing pH from 7 to 9 results in a greater fraction of ionized phenolic moieties, which have a lower reactivity than their corresponding protonated species. However, phenol is much less reactive with e_aq_^–^ (*k* = 2 × 10^7^ M^–1^ s^–1^)^16^ compared to carboxylic acids. Increasing protonation of phenols and carboxylic acids with decreasing pH lowers the DOM charge density,^61^ making the reaction of e_aq_^–^ with DOM more favorable from an electrostatic perspective. A competing effect is the impact of ionization state on DOM size. As the pH increases, electrostatic repulsion between negatively charged DOM moieties intensifies, resulting in molecular expansion^62^ and easier access to the reducible DOM moieties (e_aq_^–^ is formed in the aqueous phase upon absorption of radiation). Thus, the slight increase in *k*_DOM,e_aq_^–^_ between pH 7 and 9 is consistent with an increase in accessibility of e_aq_^–^ to reducible DOM moieties.

The ionic strength trend observed for both SRFA II and ESHA IV at pH 7 behaved according to the Brønsted-Bjerrum equation, eq 3.1, (i.e., the rate constant for like-charged reactants increases with increasing ionic strength).
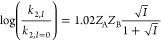
3.1In eq 3.1, *k*_2,*I*_ represents the bimolecular rate constant at ionic strength *I*, *k*_2,*I* = 0_ represents the bimolecular rate constant at infinite dilution, and *Z*_A_*Z*_B_ is the product of the charges of the reactants. We approximated *k*_2,*I*=0_ with *k*_DOM,e_aq_^–^_ values measured in 10 mM phosphate buffer (*I* = 0.02 M at pH 7). Due to the negative charge of e_aq_^–^, a higher ionic strength results in a shielding of the like-charged reactants, directly decreasing the coulombic repulsion forces and increasing reactivity with anionic species. This shielding effect was observed for both SRFA II and ESHA IV when ionic strength was increased (Figure 2B) but to slightly different extents, with *k*_DOM,e_aq_^–^_ increasing by 1.3-fold for ESHA IV and 1.5-fold for SRFA II. One possible explanation is that, at pH 7 and high ionic strength, DOM structures have expanded such that the reducible moieties are more accessible to e_aq_^–^ and some negatively charged DOM moieties have been shielded. This explanation is consistent with the abovementioned impact of increasing pH from 7 to 9 for these same isolates. Furthermore, using the Brønsted-Bjerrum equation, we calculated the *Z*_B_ value, using a (−1) charge for e_aq_^–^.^16^ The calculated *Z*_B_ values for SRFA II and ESHA IV were −0.68 and −0.44, respectively, which is much less negative than DOM charge density values reported based on other methods.^63^ One possibility is that negatively charged moieties are spatially distant from the site of e_aq_^–^ reaction. Another explanation is that increasing DOM charge impacts DOM’s three-dimensional structure and that the subsequent effect on *k*_DOM,e_aq_^–^_ is not fully captured by eq 3.1. Future research measuring *k*_DOM,e_aq_^–^_ under a greater range of pH values and ionic strength conditions could help discern among these possibilities.

Activation energies (*E*_a_) for the reaction of e_aq_^–^ with SRFA II and ESHA IV at pH 7 were calculated using the measured temperature-dependent *k*_DOM,e_aq_^–^_ values and the Arrhenius equation (eq 3.2)
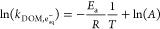
3.2where *A* is the Arrhenius pre-factor, *R* is the gas constant, and *T* is the temperature (K). Plotting ln (*k*_DOM,e_aq_^–^_) against 1000/T yields a slope −*E*_a_/*R* from which *E*_a_ was determined (Figure 2C). The activation energies for SRFA II and ESHA IV were the same within error (18.5 ± 1.4 and 17.7 ± 2.2 kJ mol^–1^, respectively), suggesting that an average *E*_a_ of 18 kJ mol^–1^ can generally be applied to assess the temperature dependence of e_aq_^–^ scavenging by DOM in ARP systems.

### 3.3 Relationships between Bimolecular Rate Constants and DOM Composition

We investigated correlations between the measured *k*_DOM,e_aq_^–^_ values and DOM physiochemical properties to provide clues to the factors governing the reactivity of DOM with e_aq_^–^. Physicochemical properties included elemental ratios (H/C and O/C), SUVA_254_, *S*_300–600_, carbon distribution from ^13^C NMR, and number-average molecular charge (*M_n_Q*). These physicochemical properties for each DOM isolate along with the respective *k*_DOM,e_aq_^–^_ values are shown in Table 1 for standard experimental conditions (pH 7.0, 10 mM phosphate buffer, 22 ± 2 °C). Elemental ratios and carbon distributions were taken from the IHSS website for each isolate’s catalog number, *M_n_Q* was taken from previous studies,^64,65^ and SUVA_254_ and *S*_300–600_ were measured in this study. Additional information about measurement and calculation of the DOM physicochemical properties and *k*_DOM,e_aq_^–^_ values under nonstandard conditions are provided in the Supporting Information (Texts S4 and S5 and Tables S7–S9).

**Table 1:** *k*_DOM,e_aq_^–^_ Values and Characterization Data for Humic Substance and NOM Isolates

sample^a^	SUVA_254_^b^,^c^ (L mg_C_^–1^ m^–1^)	*S*_300–600_^b^ (nm^–1^)	H/C^d^	O/C^d^	% aromatic^e^	% carbonyl^e^	*M*_*n*_*Q*^d^ (charge molecule^–1^)	*k*_DOM,e_aq_^–^_f (10^8^ M_C_^–1^ s^–1^)	*k*_DOM,e_aq_^–^_f (10^4^ L mg_C_^–1^ s^–1^)
ESHA IV^b^	7.4	0.0074	0.05	0.54	41	1	10.8	0.85 ± 0.02	0.71 ± 0.02
PPFA II	5.9	0.0134	0.07	0.84	39	3.6	14.7	0.66 ± 0.02	0.55 ± 0.01
PPHA I	6.1	0.0090	0.07	0.66	47	5	12.4	1.14 ± 0.04	0.95 ± 0.03
PLFA	1.2	0.0170	0.10	0.60	12	1.2	3.20	0.83 ± 0.01	0.69 ± 0.01
SRFA II^b^	4.3	0.0158	0.08	0.82	22	5	7.98	2.11 ± 0.04	1.76 ± 0.03
SRHA II^b^	5.1	0.0124	0.08	0.80	31	6	10.4	1.60 ± 0.02	1.33 ± 0.02
SRNOM II	3.2	0.0146	0.08	0.82	23	8	8.64	2.07 ± 0.05	1.73 ± 0.04
MRNOM	2.8	0.0147	0.09	0.83	19	3	9.61	0.51 ± 0.01	0.43 ± 0.01

a Experiments conducted at standard conditions of 22 ± 2 °C, pH 7.0 ± 0.1, and 10.0 mM dibasic phosphate buffer unless otherwise specified.

b IHSS catalog numbers vary for SUVA_254_ and *S*_300–600_ values. See SI Table S3.

c Values based on [DOC] calculated from isolate mass per volume normalized to IHSS percent carbon.

d Values unavailable for DOM isolates prepared in nonstandard conditions.

e Values listed here are significant figures reported on the IHSS website.^66^

f Average *k*_DOM,e_aq_^–^_ value is 1.22 ± 0.63 × 10^8^ M_C_^–1^ s^–1^ or 1.02 ± 0.53 × 10^4^ L mg_C_^–1^ s^–1^.

**Figure 3:**
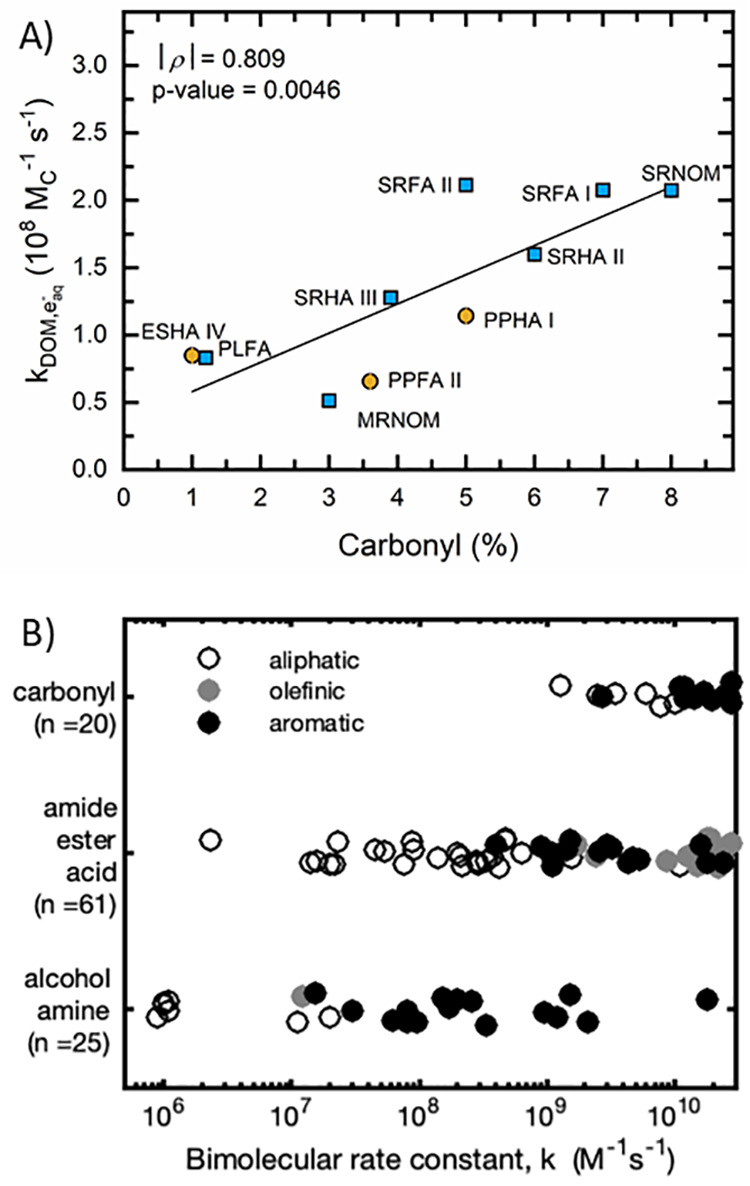
Relationships between DOM composition and e_aq_^–^ bimolecular rate constant. (A) Correlation *k*_DOM,e_aq_^–^_ and % carbonyl carbon as determined by ^13^C NMR and reported by the IHSS. Markers refer to values derived from the slope of first-order rate constants vs [DOM] (e.g., Figure 1C), and error bars refer to the standard error of the slope (majority of error bars are within markers). Marker color represents terrestrial (brown) and aquatic isolates (blue). SRNOM data represent SRNOM I for carbonyl % and SRNOM II for *k*_DOM,e_aq_^–^_. All other IHSS catalog numbers match exactly. Experiments conducted at 22 ± 2 °C, pH 7.0 ± 0.1, and 10.0 mM dibasic phosphate buffer. (B) Bimolecular rate constants between model organic compounds and e_aq_^–^ from literature sources (accessed via https://kinetics.nist.gov/solution/).^67^

Of the physicochemical properties examined, *k*_DOM,e_aq_^–^_ had the strongest relationship with the % carbonyl carbon as determined by ^13^C NMR. The positive linear correlation between *k*_DOM,e_aq_^–^_ and % carbonyl carbon (ρ = 0.809) was statistically significant (*p* = 0.0046) (Figure 3A) and is consistent with the known high reactivity of e_aq_^–^ with carbonyl compounds.^16^ For example, a sampling of literature bimolecular e_aq_^–^ rate constants for model organic compounds shows that carbonyl-containing compounds exhibit consistently higher reactivity than other functional groups (Figure 3B).

The lack of strong correlations between *k*_DOM,e_aq_^–^_ and other DOM physicochemical properties (e.g., % aromaticity, *M_n_Q*) was surprising given the known impact of charge and functional group on the reactivity of model organic compounds with e_aq_^–^. For example, we hypothesized that *k*_DOM,e_aq_^–^_ would tend to decrease with increasing DOM negative charge (*M_n_Q*), but this was not observed (see SI Figure S5). Similarly, we expected *k*_DOM,e_aq_^–^_ to be positively correlated to the electron accepting capacity,^68^ but this was also not observed (see SI Figure S5). In comparison, recent reports of bimolecular rate constants for oxidizing radicals such as SO_4_^•–^ and halogen radicals (X^•^ and X_2_^•–^) with DOM have yielded significant correlations with DOM physicochemical properties such as SUVA_254_ and electron donating capacity.^9,11,12^ Bimolecular rate constants for DOM with ^•^OH have not been described by a single parameter; rather multiple linear regression models or principal component analysis has been employed.^51,69^ Preliminary attempts were made in this study to correlate groups of 7–9 parameters, but these attempts only confirmed that % carbonyl carbon was the most significant predictor of *k*_DOM,e_aq_^–^_. These statistical analyses may prove useful in future studies on the reactivity of DOM with e_aq_^–^ but will require a larger sample set than analyzed here.

Overall, the lack of correlations between *k*_DOM,e_aq_^–^_ and DOM physicochemical properties observed herein indicates that *k*_DOM,e_aq_^–^_ is not governed by a single aspect of DOM’s composition. It is likely, however, that aquatic-based DOM will have a larger impact on e_aq_^–^ scavenging in ARP treatment due to the presence of a higher % carbonyl carbon.

### 3.4 Comparison of Organic Model Compounds and DOM Reaction with e_aq_^–^

Prior studies of radical reactions (^•^OH, SO_4_^•–^, X^•^ and X_2_^•–^; X = Cl^–^, Br^–^) with DOM have shown that measured rate constants can be reasonably well predicted using an average value of individual reacting components chosen to represent DOM composition.^7,9,11,12^ To test this hypothesis for e_aq_^–^ reaction, a set of model compounds with known e_aq_^–^ bimolecular rate constants were selected based on prior compilations for oxidizing radicals,^7,9,11,12^ and the below equation was applied (eq 3.3)
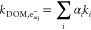
3.3where α_*i*_ and *k_i_* are the fractional contribution and bimolecular rate constant (units of M_C_^–1^s^–1^) of model organic compound *i*. α_*i*_ was varied across the three scenarios listed below to evaluate the range of possible % aromatic and % carbonyl carbon present in these isolates.

**Scenario 1**: Each model compound is set to an equal concentration resulting in 49.2% aromatic carbon and 5.3% carbonyl carbon. A 49.2% aromatic carbon is higher than aquatic isolates but only slightly higher than soil humic acids.

**Scenario 2**: The % aromatic carbon was chosen to be 20% and is partitioned equally between the aromatic compounds in the model compound set. The α_*i*_ for acetone is set to 0.07 and the remaining α_*i*_’s are distributed equally among methyl acetate, *tert*-butanol, and alanine.

**Scenario 3**: The % aromatic carbon was set to 40% and α_*i*_ for acetone is set to 0. The high aromatic % and low carbonyl % for this scenario are representative of characterization data for ESHA IV and V.

Contrary to the good agreement observed in previous studies for DOM reactions with oxidizing radicals,^7,9,11,12^ the three scenarios tested all resulted in *k*_DOM,e_aq_^–^_ values that were either at the upper end or exceeded *k*_DOM,e_aq_^–^_ values measured by pulse radiolysis (Table 2). The lower measured *k*_DOM,e_aq_^–^_ values could be the result of geometric effects (reactive e_aq_^–^ moieties are not accessible to radiolytically formed e_aq_^–^), charge impacts (DOM typically exhibits a large negative charge, which may slow down *k*_DOM,e_aq_^–^_ relative to singly charged organic compounds), or a combination of these factors. Future research is needed to discern among these possibilities.

**Table 2:** Summary of Results from Applying Eq 3.3 to Predict Hydrated Electron Rate Constants for DOM Using Model Compounds

model compound data^a^				scenario 1		scenario 2		scenario 3	
compound	formula	*k* (10^7^ M^–1^ s^–1^)	*k* (10^7^ M_C_^–1^ s^–1^)	α_*i*_	α_*i*_*k*_*i*_	α_*i*_	α_*i*_*k*_*i*_	α_*i*_	α_*i*_*k*_*i*_
phenol	C_6_H_6_O	2.00	0.33	0.11	0.037	0.04	0.01	0.08	0.03
2-hydroxybenzoate	C_7_H_6_O_3_	1000	143	0.11	15.873	0.04	5.71	0.08	11.43
benzoate	C_7_H_6_O_2_	300	42.9	0.11	4.762	0.04	1.71	0.08	3.43
benzyl alcohol	C_7_H_8_O	20	2.86	0.11	0.317	0.04	0.11	0.16	0.46
benzaldehyde	C_7_H_6_O	2400	343	0.11	38.095	0.04	13.71	0.00	0.00
acetone	C_3_H_6_O	770	257	0.11	28.519	0.07	18.74	0.02	5.13
methyl acetate	C_3_H_6_O_2_	870	2.9	0.11	0.322	0.24	0.70	0.19	0.56
*tert*-butanol	C_4_H_10_O	0.04	0.01	0.11	0.001	0.24	0.00	0.19	0.00
alanine	C_3_H_7_O_2_N	1.2	0.40	0.11	0.044	0.24	0.10	0.19	0.08
			Σ*_i_α_i_* or Σ_*i*_*α*_*i*_*k_i_*	1.00	8.8 × 10^8^ M_C_^–1^s^–1^	1.00	4.08 × 10^8^ M_C_^–1^s^–1^	1.00	2.11 × 10^8^ M_C_^–1^s^–1^

a Rate constants obtained from the NDRL/NIST solution kinetics database (kinetics.nist.gov/solution/).^67^

### 3.5 Impact of DOM on e_aq_^–^ Exposure during the UV/Sulfite ARP

The measured *k*_DOM,e_aq_^–^_ values indicate that DOM will be an important scavenger of e_aq_^–^ in ARP. In treatment technologies in which e_aq_^–^ is formed photochemically, DOM will also screen incoming UV photons from being absorbed by the e_aq_^–^ sensitizer (e.g., sulfite), thereby decreasing the rate of e_aq_^–^ formation. Both e_aq_^–^ scavenging and light screening by DOM will decrease the rate of e_aq_^–^-mediated target contaminant degradation.^45,47^ In ARP, where DOM is continuously exposed to e_aq_^–^, the light screening characteristics and e_aq_^–^ scavenging likely change over time as e_aq_^–^ reactions modify DOM structure. The *k*_DOM,e_aq_^–^_ values presented in Table 1, however, represent initial conditions before each DOM isolate has undergone transformation by e_aq_^–^.

To evaluate the impact of e_aq_^–^ exposure on DOM light screening and e_aq_^–^ scavenging, we performed an experiment in which 10 mM sodium sulfite was irradiated with low-pressure Hg vapor lamps (emitting at 254 nm) in the presence of 10 mg_C_ L^–1^ SRNOM II. UV/sulfite experiments were conducted under anerobic conditions with a reactor pH ≥ 9.5 to minimize DOM’s reaction with radical species other than e_aq_^–^.^16,45,56,57^ Even though sulfite and sulfite radicals may directly react with DOM moieties, for simplicity it was assumed that DOM reacted predominantly with e_aq_^–^ in the UV/sulfite system. However, it is not possible to categorically exclude DOM transformations by sulfite radicals. During a 24 h irradiation experiment, 20 μM chloroacetate (MCAA) was spiked at various time points to serve as an e_aq_^–^ probe as demonstrated in our previous study.^47^ The resulting first-order degradation rate constants for chloroacetate transformation were used to calculate the e_aq_^–^ concentration ([e_aq_^–^]*_t_*) and e_aq_^–^ scavenging capacity (*k*_*S*,*t*_^′^) for each chloroacetate spike time, *t*.

**Figure 4:**
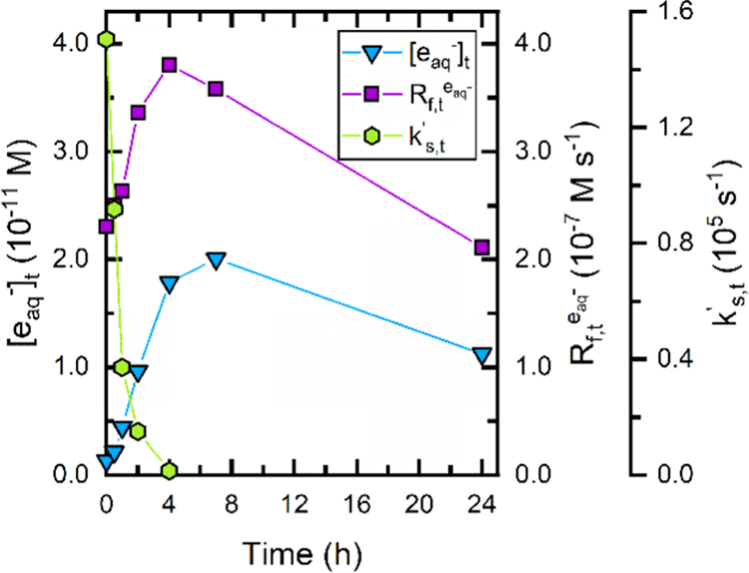
Photochemical parameters measured by chloroacetate for the UV/sulfite system in the presence of Suwanee River natural organic matter II (SRNOM II), including e_aq_^–^ concentration ([e_aq_^–^]*_t_*), formation rate (*R*_*f*,*t*_^e_aq_^–^^), and scavenging capacity (*k*_*S*,*t*_^′^). SI Text S6 explains how these parameters were calculated. Experimental conditions: 10 W low-pressure Hg lamp, pH_0_ = 9.5, 20 °C, 10 mg_C_ L^–1^ [SRNOM II]_0_, 10.4 mM [sulfite]_0_, 20 μM [MCAA]_0_ spikes, and 1.0 mM borate buffer in ultrapure water.

Results from chloroacetate spikes over a 24 h UV/sulfite experiment indicate that the light screening and e_aq_^–^ scavenging of 10 mg_C_ L^–1^ SRNOM II dissipate after ∼4 h, resulting in an [e_aq_^–^]*_t_* that peaks at ∼7 h (Figure 4, see SI Text S6 for additional details on calculations). The rate of e_aq_^–^ formation (*R*_*f*,*t*_^e_aq_^–^^), which is a function of the fraction of light absorbed by sulfite, increases from 2.3 × 10^–7^ M s^–1^ at 0 h to 3.8 × 10^–7^ M s^–1^ at 4 h. At 0 h, the solution absorbance at 254 nm was 0.49 cm^–1^, the calculated absorbance due to sulfite was 0.19 cm^–1^, and the fraction of light absorbed by sulfite was 38%. At 4 h, the fraction of light absorbed by sulfite was nearly 100%. This indicates that by ∼4 h e_aq_^–^ reactions have completely attenuated the absorbance of SRNOM II at 254 nm and that the remaining absorbance is completely attributable to sulfite (see SI Figure S6). Furthermore, the *k*_*S*,*t*_^′^value decreased rapidly during the first ∼4 h of the UV/sulfite experiment. At 0 h, the measured *k*_*S*,*t*_^′^ was 1.5 × 10^5^ s^–1^, which agrees well with the value calculated the *k*_DOM,e_aq_^–^_ value for SRNOM II in Table 1 [(10 mg_C_ L^–1^) × (1.73 × 10^4^ L mg_C_^–1^ s^–1^) = 1.73 × 10^5^ s^–1^]. By ∼4 h, the measured *k*_*S*,*t*_^′^ is consistent with the calculated *k*_*S*,*t*_^′^ of 20 μM chloroacetate (2 × 10^4^ s^–1^), indicating that the *k*_*S*,*t*_^′^ from SRNOM II has been completely abated. Taken together, the results indicate that both light screening and e_aq_^–^ scavenging decrease the [e_aq_^–^]*_t_* in the UV/sulfite system available for contaminant abatement. These impacts are anticipated to be greater at higher DOM concentrations. For example, Ren et al. demonstrated sustained light screening in the UV/sulfite system over 24 h at an Aldrich humic acid concentration of 50 mg_C_ L^–1^.^6^ This may present a challenge for e_aq_^–^-based treatment of waste streams like ion exchange resin, regeneration brine, and reverse osmosis concentrate where DOM concentrations are elevated.

## 4 Significance for Hydrated Electron-Based Contaminant Degradation

ARP have received increasing attention for the destruction of recalcitrant chemical contaminants, most notably PFAS.^45,57,70,71^ However, the role of DOM in these treatment technologies has not been adequately addressed.^45,57^ Results from this study demonstrate that e_aq_^–^ scavenging by DOM will significantly impact the rate of target contaminant degradation in ARP. We recommend that a *k*_DOM,e_aq_^–^_ value of 1.2 × 10^8^ M_C_^–1^ s^–1^ (1.0 × 10^4^ L mg_C_^–1^ s^–1^) be used to evaluate the e_aq_^–^ scavenging impact of DOM in future studies. Given that *k*_DOM,e_aq_^–^_ values vary by a factor of 4, additional research is needed to develop structure-reactivity relationships to predict e_aq_^–^ scavenging by DOM in different contexts.

Another implication of this research is that the increases in *k*_DOM,e_aq_^–^_ resulting from high ionic strength or alkaline pH, as observed in treating concentrated waste streams, are unlikely to significantly impact the efficiency of e_aq_^–^-based treatment. We showed that increasing ionic strength from 0.02 to 0.12 M or increasing pH from 5 to 9 results in only a 1.5-fold increase in *k*_DOM,e_aq_^–^_. On the other hand, increasing the DOM concentration from 10 to 100 mg_C_ L^–1^ results in a 10-fold increase in the e_aq_^–^ scavenging capacity in addition to a significant increase in UV photon screening. Thus, increases in DOM concentration in these waste streams will likely outweigh any increase in *k*_DOM,e_aq_^–^_ values that come from varying pH and ionic strength.

The temporal nature of the e_aq_^–^ formation rate, scavenging capacity, and concentration demonstrated in Figure 4 indicates that e_aq_^–^ scavenging by DOM is long-lived and has the potential to significantly impact ARP performance. The results shown in Figure 4 also raise several questions to be addressed in future research. First, we observed that the absorbance at 254 nm and e_aq_^–^ scavenging capacity of DOM were completely attenuated at ∼4 h of UV/sulfite treatment but [DOC] measured for samples collected at 2 and 24 h were the same within error of those measured at 0 h (SI Figure S9). This result indicates that the end products of e_aq_^–^ reaction with DOM are not chromophoric (do not absorb at 254 nm) and do not volatilize in our system, which was continuously sparged with nitrogen gas. Future research is needed to elucidate the composition of these products to explain the lack of change in [DOC] during UV/sulfite treatment. Second, future research should also investigate the impact of DOM in the sequential oxidation–reduction system described by Liu et al.^72^ The oxidation step, which involves the formation of ^•^OH from heat-activated persulfate, will likely be impacted by DOM given the known reactivity of ^•^OH with DOM. While mineralization of DOM in this stage may alleviate e_aq_^–^ scavenging by DOM in the subsequent reduction step, buildup of bicarbonate could negatively impact subsequent reductive treatment due to e_aq_^–^ scavenging. Third, the temporal variation in e_aq_^–^ photochemical parameters due to DOM modifications begs the question of how these parameters change in other photochemical treatment systems (i.e., UV-AOP). Although a prior study has evaluated this question and found minimal impacts under typical UV-AOP fluences (∼1000 mJ cm^–2^) using low-pressure Hg lamps,^73^ more studies are warranted.

## Supporting Information Available

List of chemicals used in this study; SUVA_254_ and spectral slope calculations from DOM absorbance measurements; kinetic data for DOM-e_aq_^–^ bimolecular rate constant determination; *M_n_Q* calculations; SRNOM II irradiation in UV/sulfite system; and measured DOC concentration during 24 h experiment (PDF)
