# Supplementary material for: Reactivity of Dissolved Organic Matter with the Hydrated Electron: Implications for Treatment of Chemical Contaminants in Water with Advanced Reduction Processes
Source: Environ Sci Technol. 2023 May 4;57(19):7634–43. doi: 10.1021/acs.est.3c00909 (PMC10862553; doi:10.1021/acs.est.3c00909)
Supplement: Supplementary file 1 — es3c00909_si_001.pdf [file es3c00909_si_001.pdf]

Supporting Information for

Reactivity of Dissolved Organic Matter with the Hydrated Electron: Implications for Treatment of Chemical Contaminants in Water with Advanced Reduction Processes

Benjamin D. Fennell,[a] Douglas Fowler,[b] Stephen P. Mezyk,[b*] and Garrett McKay[a*]

[a]Zachry Department of Civil & Environmental Engineering Texas A&M University, College Station, TX 77845

[b]Department of Chemistry and Biochemistry, California State University Long Beach, Long Beach, CA 90840

*Corresponding authors:

Garrett McKay, E-mail: gmckay@tamu.edu, Phone: 979.458.6540

Stephen P. Mezyk, E-mail: Stephen.Mezyk@csulb.edu, Phone: 562.985.4649

Number of Pages: 19

Number of Text Sections: 6

Number of Figures: 9

Number of Tables: 9

Table of Contents

Table S1. List of chemicals used in this study. 3
Table S2. List of IHSS isolates used in this study. 3
Text S1. $SUVA_{254}$ and spectral slope calculations from DOM absorbance measurements. 3
Table S3. $SUVA_{254}$ and spectral slope measured values for DOM isolates. 4
Text S2. Electron pulse radiolysis measurements. 4
Figure S1. Kinetic data for DOM-eaq- bimolecular rate constant determination. 6
Text S3. IHSS catalog number influence on DOM-e_{aq}^- bimolecular rate constants. 7
Figure S2. Kinetic data for DOM-e_{aq}^- bimolecular rate constant determination for varying IHSS catalog numbers. 7
Table S4. Bimolecular rate constants for DOM with different oxidizing radicals. 8
Figure S3. Kinetic data for DOM-eaq- bimolecular rate constant determination for varying pH and ionic strength (IS) conditions. 9
Figure S4. Kinetic data for DOM-eaq- bimolecular rate constant determination for varying temperature. 10
Text S4. M_nQ calculations. 11
Table S5. Electron accepting capacity (EAC) and number average (M_n) and weight average molecular weight (M_w) values employed in this study. 11
Table S6. Parameters for calculation of total charge density, Q (meq/gHS),[8] and the result of these calculations for each DOM isolate at the indicated pH. 12
Table S7. Elemental composition for DOM isolates.[11] 12
Table S8. Carbon distribution for DOM isolates.[12] 13
Table S9. DOM-e_{aq}^- bimolecular rate constants for varying environmental conditions. 13
Text S5. Additional physiochemical DOM properties and DOM-e_{aq}^- bimolecular rate constant correlation discussion. 13
Figure S5. Relationship between DOM-e_{aq}^- bimolecular rate constants and additional physiochemical properties. 14
Text S6. SRNOM II irradiation in UV/sulfite system. 15
Figure S6. Absorbance at 254 nm as a function of time. 17
Figure S7. Sulfite concentration as a function of time. 17
Figure S8. Fraction of UV light absorbed. 18
Figure S9. Measured DOC concentration during 24 h experiment. 18
References 19

Table S1. List of chemicals used in this study.

Chemical	Cas #	Purity
Boric acid	10043-35-3	97.97%
5,5'-dithiobis(2-nitrobenzoic acid)	69-78-3	99%
Hydrochloric acid (37%)	7647-01-0	ACS reagent
Perchloric acid (70%)	7601-90-3	ISO 9001
Potassium phosphate monobasic anhydrous	7778-77-0	99%
Potassium phosphate dibasic trihydrate	16788-57-1	99%
Sodium chloroacetate	3926-62-3	ISO 9001
Sodium hydroxide (solid pellets)	1310-73-2	≥97%
Sodium perchlorate	7601-89-0	≥98%
Sodium sulfite	7757-83-4	≥98%

Table S2. List of IHSS isolates used in this study.

IHSS Isolate	Abbreviation	Catalog Number[a]
Terrestrial		
Elliott Soil humic acid IV	ESHA IV	4S102H
Elliott Soil humic acid V	ESHA V	5S102H
Pahokee Peat fulvic acid II	PPFA II	2S103F
Pahokee Peat humic acid I	PPHA I	1S103H
Aquatic		
Pony Lake fulvic acid	PLFA	1R109F
Suwannee River fulvic acid I	SRFA I	1S101F
Suwannee River fulvic acid II	SRFA II	2S101F
Suwannee River humic acid II	SRHA II	2S101H
Suwannee River humic acid III	SRHA III	3S101H
Suwannee River NOM II	SRNOM II	2R101N
Upper Mississippi River NOM	MRNOM	1R110N

[a] IHSS catalog numbers used for pulse radiolysis experiments as described in Section 2.1 in the main manuscript. Catalog numbers listed were used for electron pulse radiolysis experiments and DOM physicochemical property correlation analysis. In a few instances, correlation analysis used a different catalog number. Deviations from catalog number listed in Table S2 are indicated subsequently below.

Text S1. $SUVA_{254}$ and spectral slope calculations from DOM absorbance measurements.

Absorbance measurements for specific ultraviolet absorbance at 254 nm ($SUVA_{254}$, L mg_C^{-1} m^{-1}) and spectral slope ($S_{300\text{-}600}$, nm^{-1}) calculations were performed on a Cary-100 UV-vis spectrophotometer (Agilent). $SUVA_{254}$ and $S_{300\text{-}600}$ were then calculated using eq. S1 and S2, respectively,

$$SUVA_{254} = \frac{A_{254} \times 100 \frac{cm}{m}}{[DOC]} \quad \text{(S1)}$$

$$A_{\lambda} = A_{\lambda_{ref}} e^{-S(\lambda - \lambda_{ref})} \quad \text{(S2)}$$

where A_{254} is the absorbance at 254 nm wavelength (cm^{-1}), [DOC] is the concentration of dissolved organic carbon (mg_C^{-1} L) calculated based on the measured mass of isolate and the % m/m carbon, a_{λ} is the absorbance (cm^{-1}) at wavelength λ is the wavelength, and λ_{ref} is the reference wavelength (300 nm).[1] Spectral slope was calculated from eq. S2 using the exponential fitting function in Excel. Table S3 lists the $SUVA_{254}$ and $S_{300\text{-}600}$ values for each DOM isolate.

Table S3. $SUVA_{254}$ and spectral slope measured values for DOM isolates.

DOM Isolate[a]	Catalog Number	$SUVA_{254}$ (L mg_C^{-1} m^{-1})	$S_{300\text{-}600}$ (nm^{-1})
SRNOM II	2R101N	3.2	0.0146
MRNOM	1R110N	2.8	0.0147
SRFA III	3S101F	4.3	0.0158
PPFA II	2S103F	5.9	0.0134
SRHA III	3S101H	5.1	0.0124
PPHA I	1S103H	6.1	0.0090
ESHA V	5S102H	7.4	0.0074
PLFA	1R109F	1.2	0.0170
SRFA III, pH 5	3S101F	4.1	0.0156
SRFA III, pH 9	3S101F	4.3	0.0160
ESHA V, pH 9	5S102H	7.3	0.0073
SRFA III, 0.1 M ionic strength	3S101F	4.6	0.0153
ESHA V, 0.1 M ionic strength	5S102H	7.2	0.0074

[a] Solutions prepared at standard conditions of 20 ± 2 °C, pH 7.0 ± 0.1, and 10.0 mM dibasic phosphate buffer unless otherwise specified.

Text S2. Electron pulse radiolysis measurements.

The radiolysis of water initiated by a fast electron pulse produces several radical and molecular species as observed in eq. S3,[2]

$$H_2O -\backslash\backslash\backslash\backslash\rightarrow [0.28]HO^\bullet + [0.06]H^\bullet + [0.27]e_{aq}^- + [0.05]H_2 + [0.07]H_2O_2 + [0.27]H_3O^+ \quad (S3)$$

where the bracketed numbers are the *G*-value or yield (µM J^{-1}) of species produced at 10^{-7} s after irradiation. The transient decay kinetics of e_{aq}^- were monitored at 720 nm using transient absorption spectroscopy. No effort was undertaken to isolate e_{aq}^- by adding *t*-butanol because we were concerned that *t*-butanol could impact DOM macrostructures. Although the presence of other radical species like $^\bullet$OH can impact the e_{aq}^- lifetime in solution, this is not of concern here for two reasons. First, although $^\bullet$OH will react with e_{aq}^-, the rate will be the same in all solutions because the nominal pulse intensity is the same in each experiment and the rate is overall low due to the low concentration of each radical species (ca. 2-4 µM for each radical). Thus, e_{aq}^- reaction with $^\bullet$OH will be a constant component of the background e_{aq}^- decay. Second, there will be significant scavenging of the $^\bullet$OH radical by the DOM itself,[3] which will further reduce the $^\bullet$OH radical free concentration. Also, it is unlikely that e_{aq}^- would react with a moiety in DOM oxidized by $^\bullet$OH in the same µs timescale.

Figure S1 shows first-order e_{aq}^- decay constants derived from transient absorption data plotted against DOM concentration. The slope of each line represents the k_{DOM,e_{aq}^-}. The y-intercept represents any e_{aq}^- scavengers other than DOM present in the background anerobic water. For example, H^+ will be an important e_{aq}^- scavenger for experiments conducted at acidic pH given the high bimolecular rate constant of 2.3×10^{10} $M^{-1}s^{-1}$.[4] Given that the background solvent's scavenging capacity remains the same, the change in first order e_{aq}^- decay constants are determined exclusively by changes in the DOM concentration. H^+ in this instance serves as a constant background e_{aq}^- scavenger under all experimental conditions.

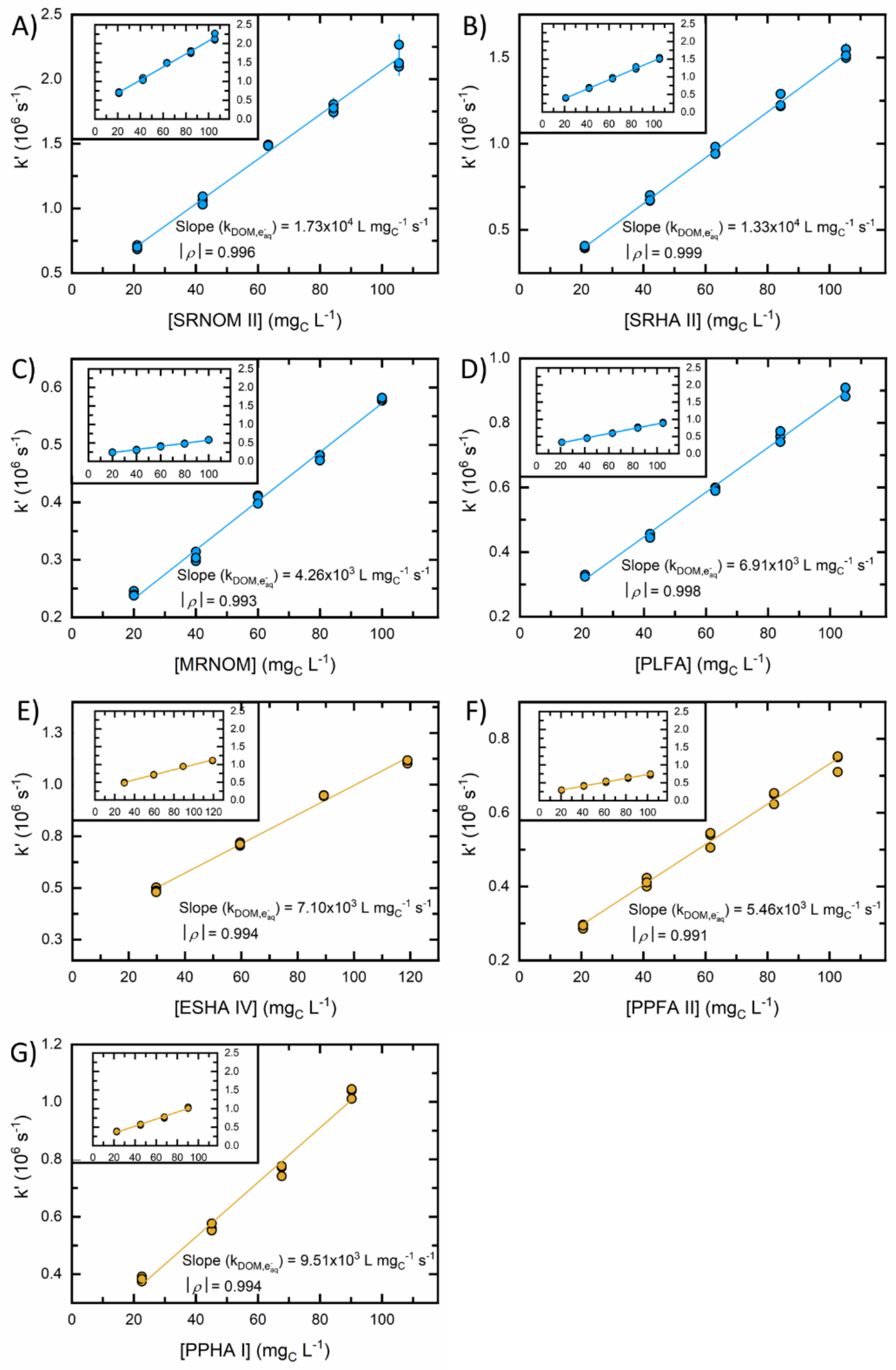

Figure S1. Kinetic data for DOM-eaq- bimolecular rate constant determination. Line represents a linear fit to the data using the least squares method with the slope reported as the bimolecular rate constant ($k_{DOM,e^-_{aq}}$). Aquatic DOM isolates (blue color) analyzed include A) SRNOM II, B) SRHA II, C) MRNOM, D) PLFA, and SRFA II (Figure 1C, main manuscript). Terrestrial DOM isolates (brown color) analyzed include E) ESHA IV, F) PPFA II, and G) PPHA I. Markers represent pseudo-first-order rate constants determined from transient e_{aq}^- decay data and error bars represent uncertainty of the fitted data (majority of error bars are within markers). Insets show data plotted on equivalent y-axis to compare between samples. Experiments conducted at pH 7.0 ± 0.1, 22 ± 2 ºC, and 10.0 mM dibasic phosphate buffer.

Text S3. IHSS catalog number influence on DOM-e_{aq}^- bimolecular rate constants.

We assessed the impact of different IHSS catalog numbers on k_{DOM,e_{aq}^-} using the multiple samples available for SRFA and SRHA. As seen in Figures S2A-S2B, SRFA I (1S101F) and SRFA II (2S101F) fall within the error bounds of each other, meaning that SRFA I and SRFA II have the same k_{DOM,e_{aq}^-}. However, for SRHA, we observed a 18% difference between SRHA II (2S101H) and SRHA III (3S101H), as seen in Figures S2C-S2D.

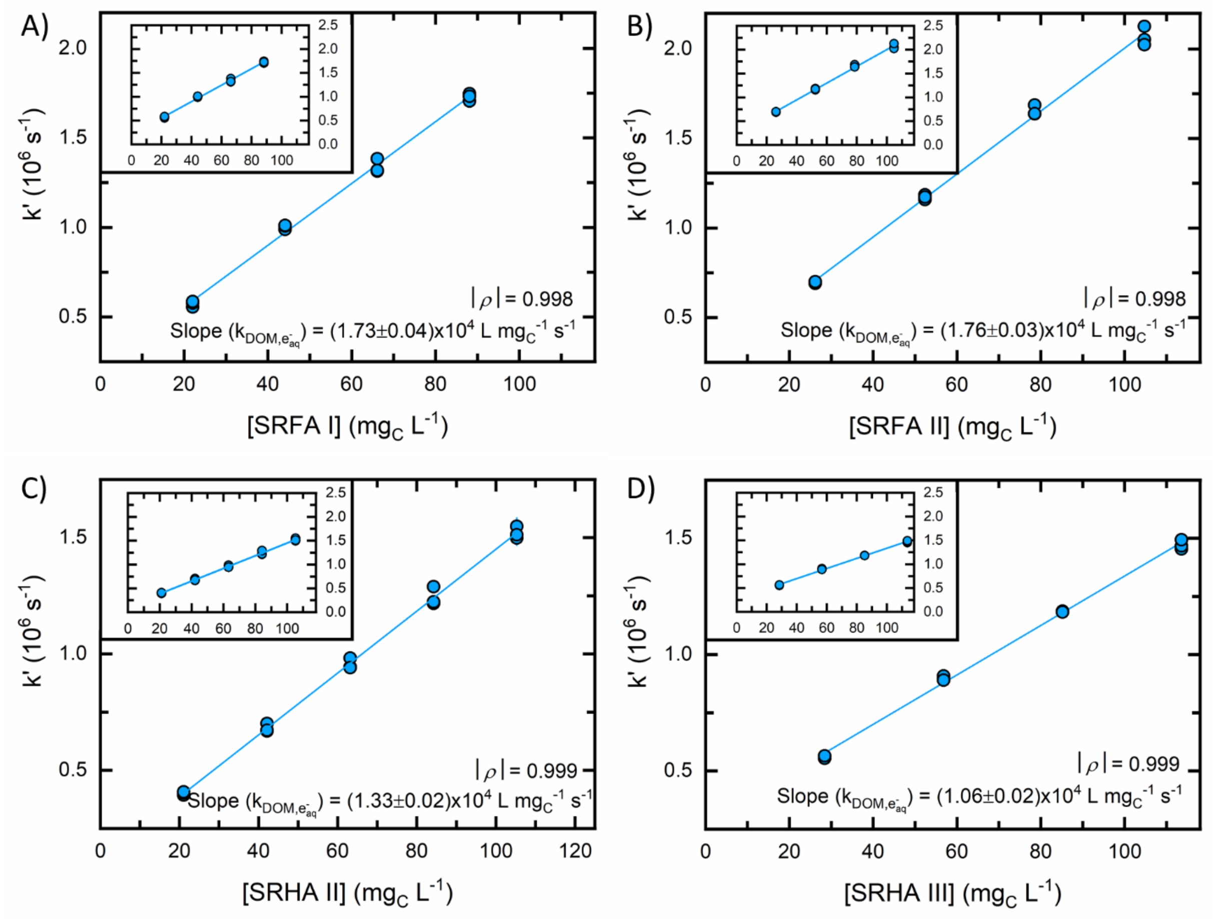

Figure S2. Kinetic data for DOM-e_{aq}^- bimolecular rate constant determination for varying IHSS catalog numbers. Line represents a linear fit to the data using the least squares method with the slope reported as the bimolecular rate constant (k_{DOM,e_{aq}^-}). Aquatic DOM isolates (blue color) analyzed include A) SRFA I, B) SRFA II, C) SRHA II, and D) SRHA III. Markers represent pseudo-first-order rate constants determined from transient e_{aq}^- decay data and error bars represent uncertainty of the fitted data (majority of error bars are within markers). Insets show data plotted on equivalent y-axis to compare between samples. Experiments conducted at pH 7.0 ± 0.1, 10.0 mM dibasic phosphate buffer, and controlled temperature (e.g, 25 °C for A) and D), 20 °C for B), and 22 °C for C)).

Table S4. Bimolecular rate constants for DOM with different oxidizing radicals.

**DOM Isolate**[a]	$k_{DOM,\bullet OH}$ (10^8 M_C^{-1} s^{-1})[3, 5]	$k_{DOM,SO_4^{\bullet-}}$ (10^7 M_C^{-1} s^{-1})[6]	$k_{DOM,Cl^\bullet}$ (10^8 M_C^{-1} s^{-1})[7]	$k_{DOM,Cl_2^{\bullet-}}$ (10^7 M_C^{-1} s^{-1})[7]	$k_{DOM,CO_3^{\bullet-}}$ (10^6 M_C^{-1} s^{-1})[8]
SRFA	1.39±0.16[3]				
SRFA	1.87±0.07[3]				
SRFA	1.55±0.04[3]				
Saguaro Lake Hydrophobic Acid	1.73±0.04[3]				
Saguaro Lake Transphilic Acid	1.45±0.02[3]				
Saguaro Lake Hydrophobic Neutral	2.18±0.13[3]				
Nogales WWTP Hydrophobic Neutral	1.72±0.13[3]				
Nogales WWTP Transphilic Neutral	4.53±0.54[3]				
Nogales WWTP Transphilic Acid	3.63±0.31[3]				
SRFA I	2.08±0.18[5]				
ESHA	1.21±0.12[5]				
Leonardite humic acid (LHA)	6.47±0.26[5]	3.68±0.34	5.20±0.27	3.57±0.53	
PLFA	6.9±0.82[5]				
SRHA II	10.36±0.02[5]				
SRNOM II		1.97±0.21	4.12±0.32	1.64±0.35	1.25±0.07
Nordic Lake NOM (NLNOM)		2.36±0.18	4.50±0.30	1.31±0.17	1.40±0.07
MRNOM		1.39±0.12	5.93±0.29	2.18±0.18	
SRFA II		2.78±0.24	6.60±0.63	2.27±0.21	1.74±0.06
PPFA II		3.07±0.26	10.2±0.80	2.75±0.53	
ESFA V		1.80±0.15			
Nordic Lake fulvic acid (NLFA)		3.22±0.25	10.4±1.02	2.93±0.36	
SRHA III		2.76±0.22			
PPHA I		2.73±0.19	4.26±0.42	2.61±0.48	
ESHA IV		3.48±0.28	5.30±0.49	2.8±0.61	
ESHA V		2.18±0.17			
Saguaro Lake HPOA (SL-HPOA)		0.87±0.09	5.64±0.33	0.46±0.09	
Saguaro Lake TPIA (SL-TPIA)		0.93±0.11	8.30±0.40	0.65±0.12	
Nogales WWTP EfOM (EfOM-1)		0.75±0.06	8.42±0.51	1.55±0.29	
Guangzhou WWTP EfOM (EfOM-2)		0.72±0.03			
Microcystis aeruginosa IOM (TLAOM)		0.64±0.05			
Anabaena IOM (YXAOM)		0.70±0.06			
Chlorella IOM (XQAOM)		1.02±013			

[a] EfOM = effluent organic matter; HPOA = hydrophobic acid; TPIA = transphilic acid; IOM = intracellular organic matter.

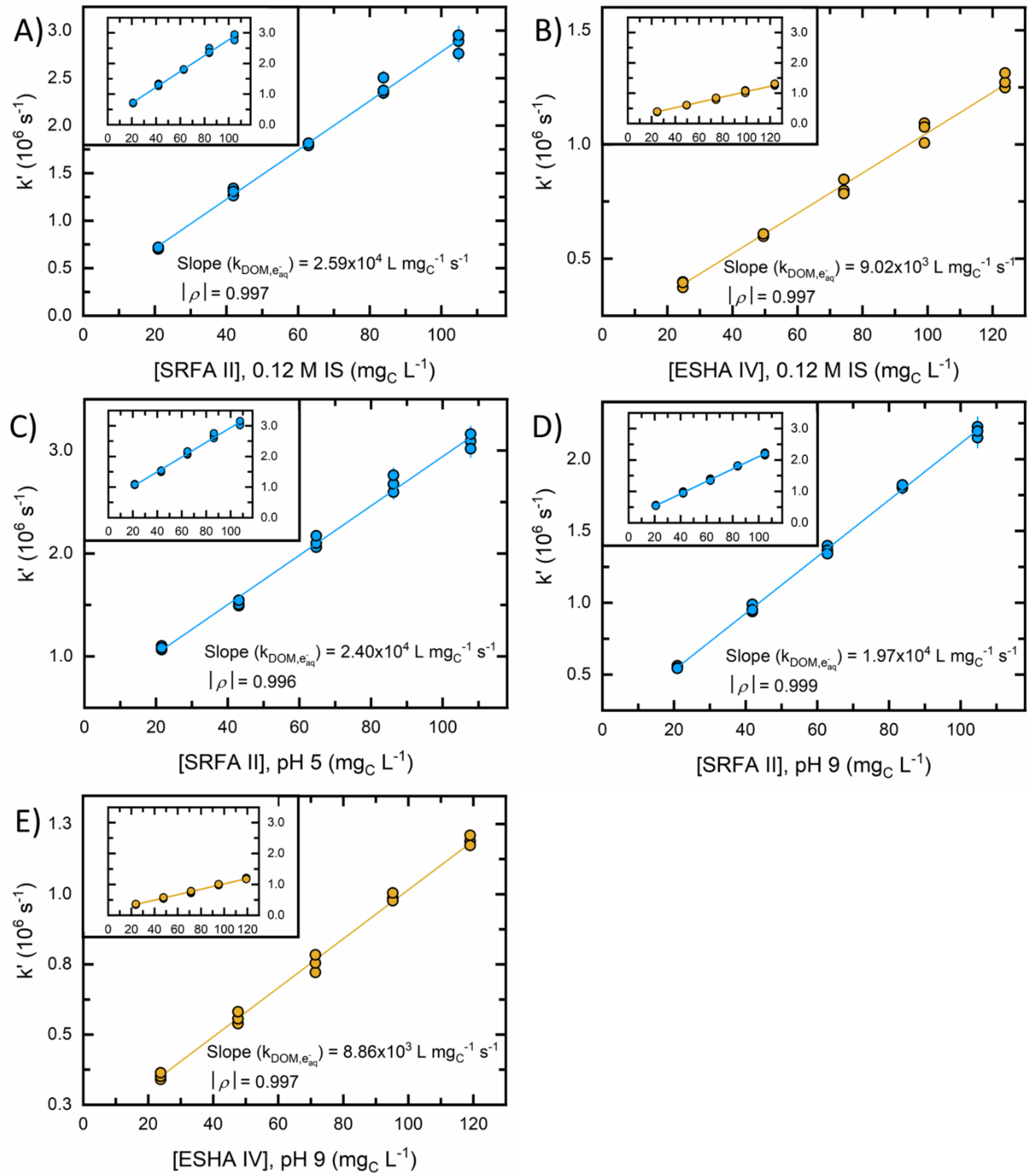

Figure S3. Kinetic data for DOM-e_{aq}^- bimolecular rate constant determination for varying ionic strength (IS) (A-B) and pH conditions (C-E). Line represents a linear fit to the data using the least squares method with the slope reported as the bimolecular rate constant (k_{DOM,e_{aq}^-}). SRFA II (blue color) and ESHA IV (brown color) utilized as representative DOM isolates. Markers represent pseudo-first-order rate constants determined from transient e_{aq}^- decay data and error bars represent uncertainty of the fitted data (majority of error bars are within markers). Insets show data plotted on equivalent y-axis to compare between samples. Experiments conducted at pH 7.0 ± 0.1, 22 ± 2 ºC, and 10.0 mM dibasic phosphate buffer unless otherwise specified.

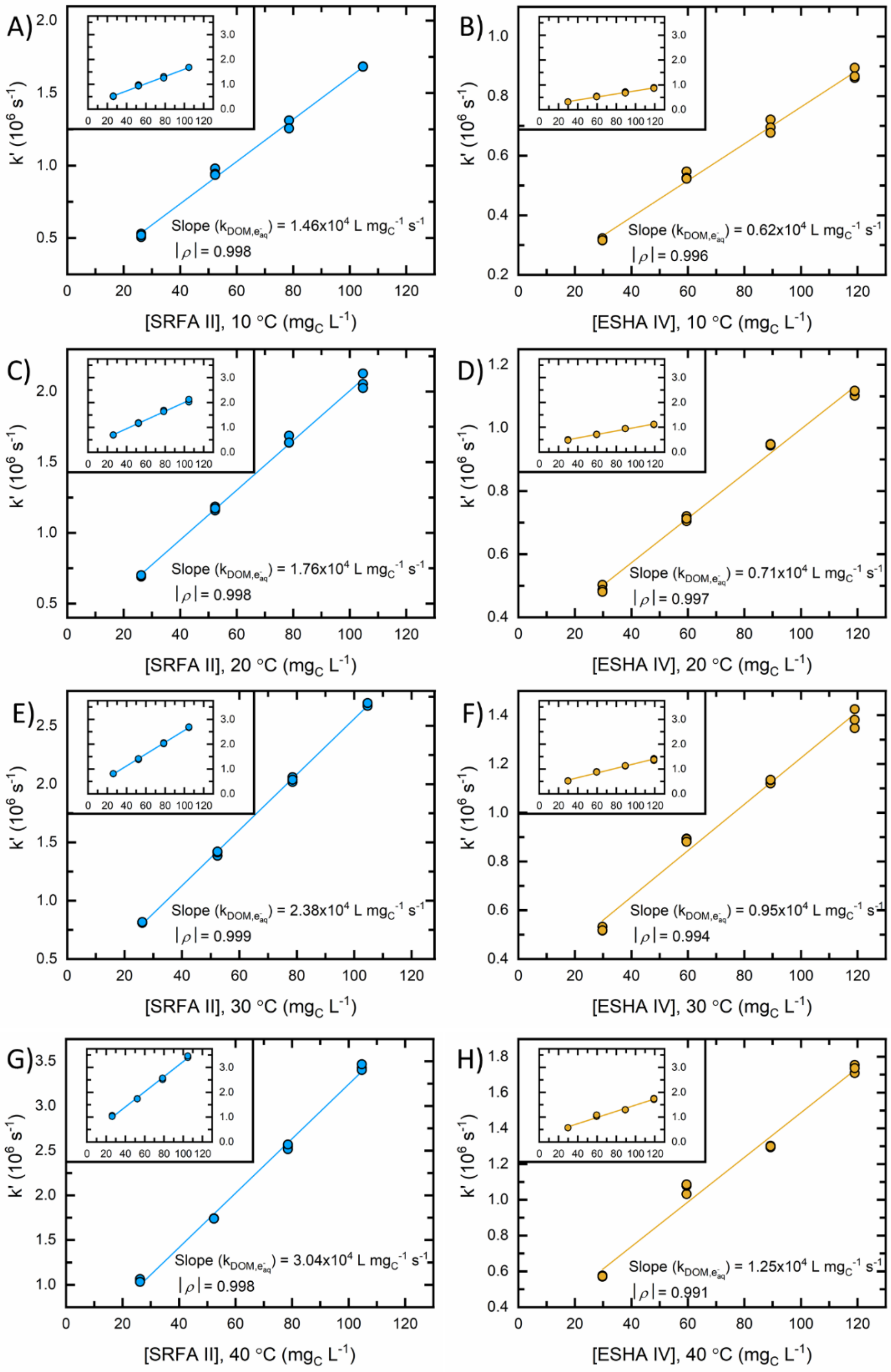

Figure S4. Kinetic data for DOM-e_{aq}^- bimolecular rate constant determination for varying temperature (A-H). Line represents a linear fit to the data using the least squares method with the slope reported as the bimolecular rate constant (k_{DOM,e_{aq}^-}). SRFA II (blue color) and ESHA IV (brown color) utilized as representative DOM isolates. Markers represent pseudo-first-order rate constants determined from transient e_{aq}^- decay data and error bars represent uncertainty of the fitted data (majority of error bars are within markers). Insets show data plotted on equivalent y-axis to compare between samples. Experiments conducted at pH 7.0 ± 0.1, 22 ± 2 ºC, and 10.0 mM dibasic phosphate buffer unless otherwise specified.

Text S4. M_nQ calculations.

The number average molecular charge (M_nQ) was calculated as a product of the number average molecular weight (M_n, Table S5) and the total charge density (Q, Table S6). Q was calculated by eq. S4,

$$Q = \left(\left(\frac{Q_1}{1 + (K_1[H^+]^{1/n_1})}\right) + \left(\frac{Q_2}{1 + (K_2[H^+]^{1/n_2})}\right)\right)\left(\% \frac{m}{m} carbon\right) \quad \text{(S4)}$$

where Q_1 and Q_2 are the charge densities, K_1 and K_2 are the average equilibrium constants, and n_1 and n_2 are the empirical parameters from pH titration data.[9] The % m/m refers to the conversion of the charge density from a per gram humic substance (gHS) to per gram carbon (gC) basis.

Table S5. Electron accepting capacity (EAC) and number average (M_n) and weight average molecular weight (M_w) values employed in this study.

**DOM Isolate**	**Catalog Number**	**EAC[a] (μmol e^- gHS^{-1})**	**M_n[b] (Da)**	**M_w[b] (Da)**
ESHA IV	4S102H	1962	2399	16489
PPFA II	2S103F	992	2310	6863
PPHA I	1S103H	1684	2591	15359
PLFA	1R109F	493	909	3519
SRFA II	2S101F	671	1436	5278
SRHA II	2S101H	962	2329	9159
SRNOM II	2R101N	653	1748	6306
MRNOM	1R110N	750	1611	4733

[a] Electron accepting capacity (EAC) values from Aeschbacher *et al.*,[10] [b] M_n and M_w values from Li and McKay.[11]

Table S6. Parameters for calculation of total charge density, Q (meq/gHS),[9] and the result of these calculations for each DOM isolate at the indicated pH.

DOM Isolate	Catalog Number	Q_1	$Log\ K_1$	n_1	Q_2	$Log\ K_2$	n_2	Q^b (meq/gHS)
ESHA I[a]	1S102H	8.90	4.36	3.16	0.85	9.80	1.00	4.51
ESHA I, pH 9[a]	1S102H	8.90	4.36	3.16	0.85	9.80	1.00	5.07
PPFA I[a]	1S103F	14.22	3.99	3.33	0.76	9.57	1.00	6.38
PPHA I	1S103H	1.91	9.64	4.22	3.20	0.94	9.86	4.79
PLFA	1R109F	6.91	4.52	1.92	1.43	9.48	1.77	3.48
SRFA II	2S101F	5.0	11.66	3.76	3.24	2.05	9.84	5.56
SRFA II, pH 5	2S101F	5.0	11.66	3.76	3.24	2.05	9.84	4.22
SRFA II, pH 9	2S101F	5.0	11.66	3.76	3.24	2.05	9.84	6.18
SRHA II	2S101H	9.74	4.35	3.30	4.48	10.44	1.73	4.45
SRNOM II	2R101N	11.20	4.16	3.44	1.60	9.99	1.03	4.94
MRNOM	1R110N	12.51	3.47	2.69	0.91	10.00	1.00	5.96

[a] Information not available for IHSS catalog numbers used in pulse radiolysis experiments (see Section 2.1 in the main manuscript). [b] Calculations conducted at pH 7 unless otherwise specified.

Table S7. Elemental composition for DOM isolates.[12]

DOM Isolate	Catalog Number	Carbon (%)	Hydrogen (%)	Oxygen (%)	Nitrogen (%)
ESHA IV	4S102H	59.51	3.20	32.16	3.90
ESHA V	5S102H	58.80	3.68	32.90	4.22
PPFA II	2S103F	51.31	3.53	43.32	2.34
PPHA I	1S103H	56.37	3.82	37.34	3.69
PLFA	1R109F	52.47	5.39	31.38	6.51
SRFA I	1S101F	52.44	4.31	42.20	0.72
SRFA II	2S101F	52.34	4.36	42.98	0.67
SRFA III	3S101F	53.30	3.98	41.81	0.66
SRHA II	2S101H	52.63	4.28	42.04	1.17
SRHA III	3S101H	54.59	3.90	40.03	1.50
SRNOM II	2R101N	50.70	3.97	41.48	1.27
MRNOM	1R110N	49.98	4.61	41.40	2.36

Table S8. Carbon distribution for DOM isolates.[13]

DOM Isolate	Catalog Number	Carbonyl (%)	Carboxyl (%)	Aromatic (%)	Acetal (%)	Heteroaliphatic (%)	Aliphatic (%)
ESHA IV	4S102H	1	11	41	6	14	27
ESHA V	5S102H	1	14.8	48.3	5.1	9.6	16.2
PPFA II	2S103F	3.6	18.7	39	6	10.9	18.4
PPHA I	1S103H	5	20	47	4	5	19
PLFA	1R109F	1.2	17	12	0.2	8.4	61
SRFA I	1S101F	7	20	24	5	11	33
SRFA II	2S101F	5	17	22	6	16	35
SRFA III	3S101F	4.2	15.6	28.9	8.1	13.3	27.4
SRHA II	2S101H	6	15	31	7	13	29
SRHA III	3S101H	3.9	12.8	35.3	8.9	13.4	23.9
SRNOM I	1R101N	8	20	23	7	15	27
MRNOM	1S110N	3	14	19	7	20	37

Table S9. DOM-e_{aq}^- bimolecular rate constants for varying environmental conditions.

DOM Isolate[a]	$SUVA_{254}$ (L mg_C^{-1} m^{-1})	k_{DOM,e_{aq}^-} (10^8 M_C^{-1} s^{-1})	k_{DOM,e_{aq}^-} (10^4 L mg_C^{-1} s^{-1})
Terrestrial			
ESHA IV, pH 9	7.3	1.06±0.02	0.89±0.02
ESHA IV, 0.1 M ionic strength	7.2	1.08±0.02	0.90±0.02
ESHA IV, 10 ºC		0.74±0.02	0.62±0.02
ESHA IV, 30 ºC		1.14±0.04	0.95±0.03
ESHA IV, 40 ºC		1.50±0.06	1.25±0.05
Aquatic			
SRFA II, pH 5	4.1	2.88±0.07	2.40±0.06
SRFA II, pH 9	4.3	2.37±0.03	1.97±0.02
SRFA II, 0.1 M ionic strength	4.6	3.11±0.07	2.59±0.06
SRFA II, 10 ºC		1.75±0.04	1.46±0.03
SRFA II, 30 ºC		2.86±0.02	2.38±0.02
SRFA II, 40 ºC		3.65±0.07	3.04±0.05

[a] Experiments conducted at standard conditions of 22 ± 2 ºC, pH 7.0 ± 0.1, and 10.0 mM dibasic phosphate buffer unless otherwise specified. IHSS catalog numbers for $SUVA_{254}$ values are different than those used for pulse radiolysis, as explained in main manuscript Section 2.1.

Text S5. Additional physiochemical DOM properties and DOM-e_{aq}^- bimolecular rate constant correlation discussion.

Figure S5 relationships between DOM-e_{aq}^- bimolecular rate constants and additional DOM physicochemical properties, including the H/C ratio (S2A), *Q* (S2B), M_n (S2C), M_nQ (S2D), $SUVA_{254}$ (S2E), and $S_{300\text{-}600}$ (S3F). See Section 3.3 in the main manuscript for additional explanation.

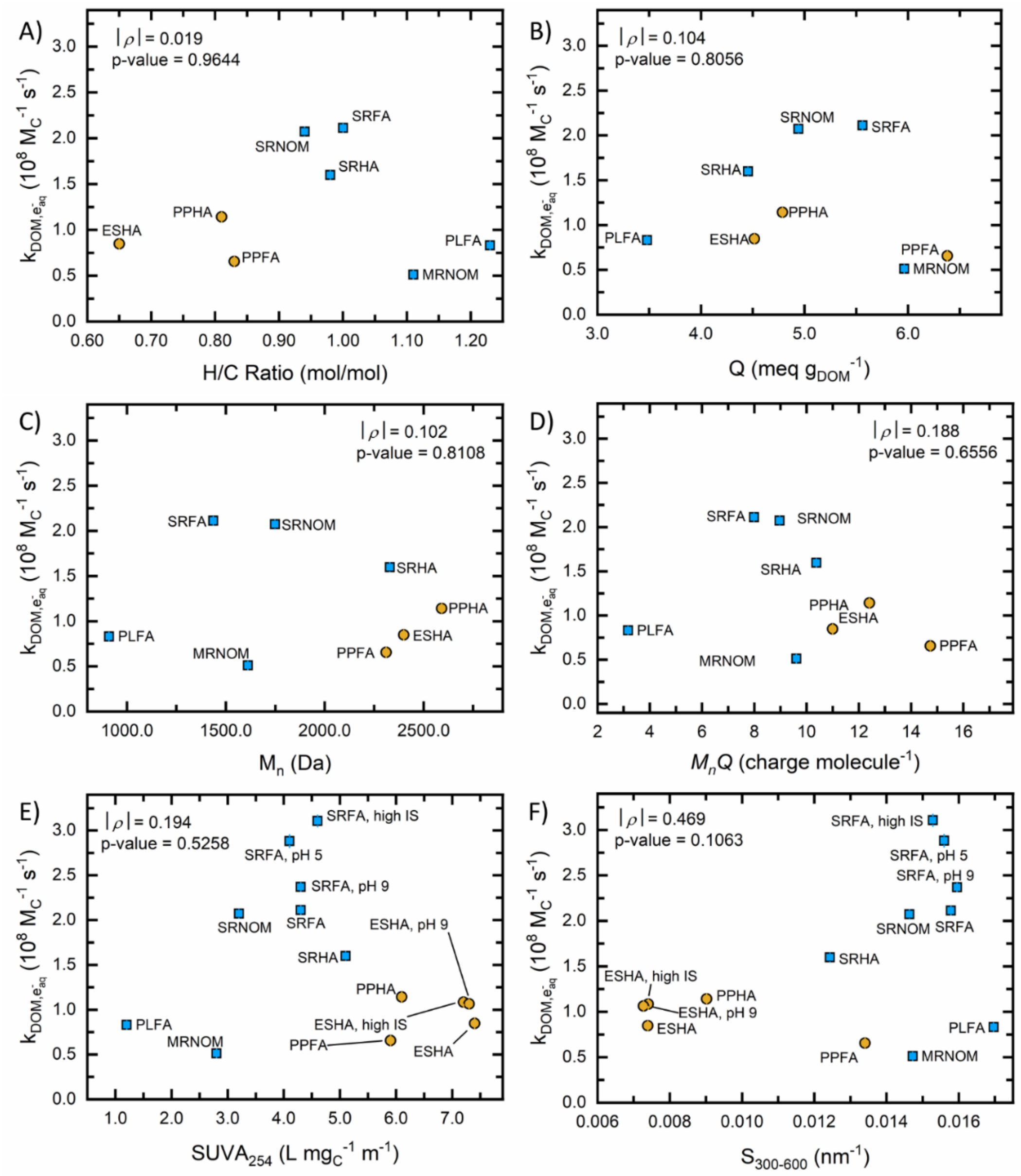

Figure S5. Relationship between DOM-e_{aq}^- bimolecular rate constants and additional physiochemical properties. Linear correlations of DOM-e_{aq}^- bimolecular rate constants (k_{DOM,e_{aq}^-}) and physiochemical properties for terrestrial DOM isolates (brown color) and aquatic DOM isolates (blue color). Physicochemical properties include A) H/C ratio, B) Q, C) M_n, D) M_nQ, E) $SUVA_{254}$, and F) $S_{300\text{-}600}$. IHSS catalog numbers may vary for physiochemical properties (see Table S6-S9 footnotes). Error bars represent the standard error of the bimolecular rate constant. Experiments conducted at pH 7.0 ± 0.1, 22 ± 2 ºC, and 10.0 mM dibasic phosphate buffer unless otherwise specified.

Text S6. SRNOM II irradiation in UV/sulfite system.

To explore the time-based e_{aq}^- scavenging impact of DOM, we conducted a 24 h, UV photolysis experiment with 10 mg_C L^{-1} SRNOM II, 10.4 mM sulfite, 20 μM chloroacetate (MCAA) spikes, and 1.0 mM borate buffer at pH 9.5 and 20 ºC. The change in total absorbance at 254 nm relative to SRNOM II absorbance is shown in Figure S6. SRNOM II absorbance was calculated by subtracting the absorbance of sulfite (determined by the Beer-Lambert law, a measured sulfite concentration (see Figure S7), and a molar absorption coefficient of sulfite at 254 nm as 18.14 M^{-1} cm^{-1})[14] and MCAA (measured spectrophotometrically for 20 μM MCAA at pH 9.5) from the total absorbance. Interestingly, the absorbance of SRNOM II decreases within the first 4 h of irradiation, providing initial evidence that the SRNOM II-e_{aq}^- scavenging impact may change over time.

Key kinetic parameters were calculated to investigate and quantify the impact of e_{aq}^- scavenging by SRNOM II using the $R_{e-,UV}$ method we previously developed.[15] Briefly, a probe compound, MCAA, was spiked into solution at key time points throughout our 24 h experiment to quantify the time-based e_{aq}^- concentration, $[e_{aq}^-]_t$. The total e_{aq}^- scavenging capacity (e_{aq}^- scavenging capacity of the water matrix, $k'_{S,t}$, plus e_{aq}^- scavenging capacity of MCAA, $k'_{MCAA,t}$, (s^{-1})) at time t was calculated as the rate of e_{aq}^- formation, $R_{f,t}^{e_{aq}^-}$ (M s^{-1}), divided by the $[e_{aq}^-]_t$ as shown in eq. S5.

$$[e_{aq}^-]_t = \frac{R_{f,t}^{e_{aq}^-}}{k'_{S,t} + k'_{MCAA,t}} \quad \text{(S5)}$$

Figure 4 (main manuscript) shows a plot of the $[e_{aq}^-]_t$, $R_{f,t}^{e_{aq}^-}$, and $k'_{S,t}$ over our 24 h experiment. Notably, $[e_{aq}^-]_t$ increases as the $k'_{S,t}$ decreases within the first 4 h. Due to our experimental system setup and absorbance data presented in Figure S6, the primary e_{aq}^- scavenger within the first 4 h is

SRNOM II. There is thus a direct correlation to an increase in $[e_{aq}^-]_t$ when SRNOM II is reduced by e_{aq}^- within the first 4 h.

Another method to calculate $k'_{S,t}$ is shown in eq. S6,

$$k'_{S,t} = \sum_i k_{S_i,e_{aq}^-}[S_i]_t \tag{S6}$$

where k_{S_i,e_{aq}^-} is the e_{aq}^- bimolecular rate constant of the scavenger and $[S_i]_t$ is the scavenger concentration listed at time *t*. If we assume that nearly all $k'_{S,t}$ is attributed to SRNOM II at time 0, we can compute $k'_{S,t}$ as the product of $k_{SRNOM\ II,e_{aq}^-}$ (1.73×10^4 L mg_C^{-1} s^{-1}) times 10 mg_C L^{-1} $[SRNOM\ II]_0$, or 1.73×10^5 s^{-1}. This value falls within 12% of our $k'_{S,t}$ measured by the $R_{e-,UV}$ method.

Furthermore, the impact of DOM chromophores competing for UV photons can be observed by closely looking at the $R_{f,t}^{e_{aq}^-}$ data in Figure 4 (main manuscript). As SRNOM II is reduced by e_{aq}^-, the chromophoric or light absorbing portion of DOM also is reduced, allowing more of the incoming UV photons to be absorbed by the UV sensitizer sulfite. The $R_{f,t}^{e_{aq}^-}$ is primarily driven in this case by the fraction of UV light absorbed by the sensitizer. As shown in Figure S8, the fraction of UV light absorbed by sulfite increases to a maximum around 4 h, correlating with the maximum $R_{f,t}^{e_{aq}^-}$. From 4-24 h, sulfite serves as the main e_{aq}^- scavenger in the UV system, resulting in an abrupt decrease in sulfite concentration throughout the remainder of the experiment (Figure S7). In this manner, SRNOM II serves as both an e_{aq}^- scavenger and UV light absorber within the first 4 h of our experiment. It is anticipated that higher concentrations of DOM or other DOM types with more chromophores will have an even more pronounced impact on e_{aq}^- scavenging and competition for UV light, directly impacting UV-ARP treatment of contaminants in natural source waters.

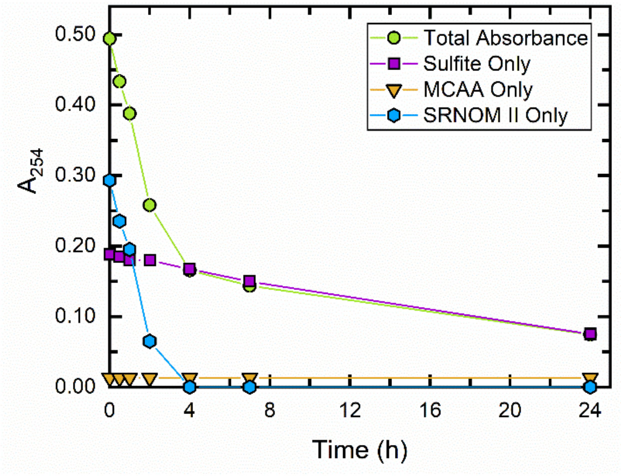

Figure S6. Absorbance at 254 nm as a function of time. Experimental conditions include 10 W low-pressure Hg lamp, pH_0 = 9.5, 20 ºC, 10 mg_C L^{-1} $[SRNOM\ II]_0$, 10.4 mM $[sulfite]_0$, 20 µM $[MCAA]_0$ spikes, and 1.0 mM borate buffer in ultra-pure water. A_{254} contributed by SRNOM II was calculated by subtracting the A_{254} by sulfite and A_{254} by MCAA from the total absorbance.

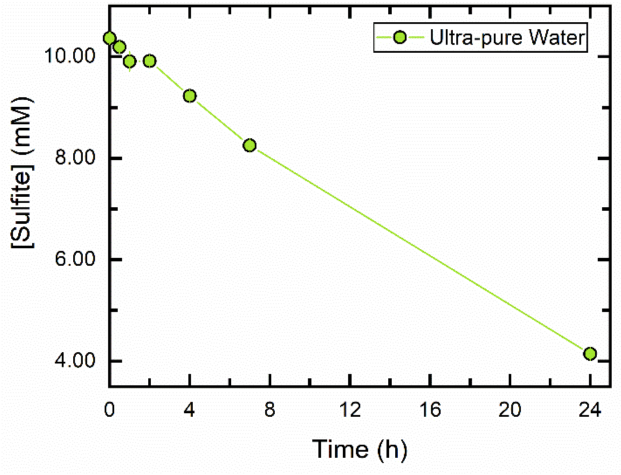

Figure S7. Sulfite concentration as a function of time. Experimental conditions include 10 W low-pressure Hg lamp, pH_0 = 9.5, 20 ºC, 10 mg_C L^{-1} $[SRNOM\ II]_0$, 10.4 mM $[sulfite]_0$, 20 µM $[MCAA]_0$ spikes, and 1.0 mM borate buffer in ultra-pure water.

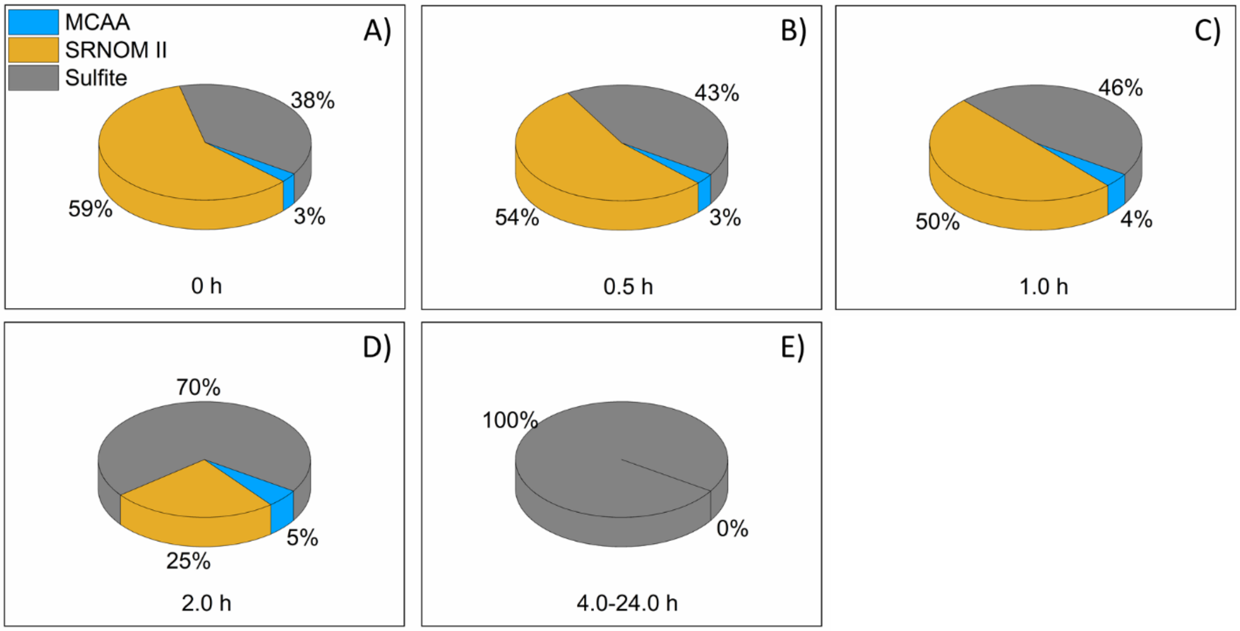

Figure S8. Fraction of UV light absorbed. Experimental conditions include 10 W low-pressure Hg lamp, $pH_0 = 9.5$, 20 ºC, 10 $mg_C\ L^{-1}$ $[SRNOM\ II]_0$, 10.4 mM $[sulfite]_0$, 20 µM $[MCAA]_0$ spikes, and 1.0 mM borate buffer in ultra-pure water.

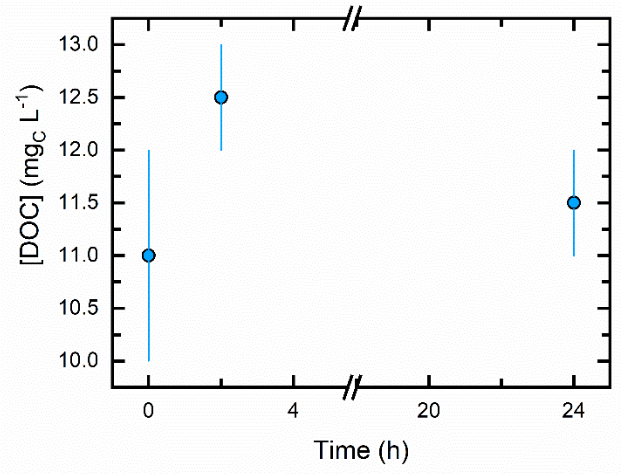

Figure S9. Measured DOC concentration during 24 h experiment**.** Experimental conditions include 10 W low-pressure Hg lamp, $pH_0 = 9.5$, 20 ºC, 10 $mg_C\ L^{-1}$ $[SRNOM\ II]_0$, 10.4 mM $[sulfite]_0$, 20 µM $[MCAA]_0$ spikes, and 1.0 mM borate buffer in ultra-pure water.

References

1. Twardowski, M. S.; Boss, E.; Sullivan, J. M.; Donaghay, P. L., Modeling the Spectral Shape of Absorption by Chromophoric Dissolved Organic Matter. *Marine Chemistry* **2004,** *89* (1-4), 69-88.
2. Buxton, G. V.; Greenstock, C. L.; Helman, W. P.; Ross, A. B., Critical Review of Rate Constants for Reactions of Hydrated Electrons, Hydrogen Atoms and Hydroxyl Radicals ($\cdot OH/\cdot O^{-}$) in Aqueous Solution. *Journal of Physical and Chemical Reference Data* **1988,** *17* (2), 513-886.
3. Westerhoff, P.; Mezyk, S. P.; Cooper, W. J.; Minakata, D., Electron Pulse Radiolysis Determination of Hydroxyl Radical Rate Constants with Suwannee River Fulvic Acid and Other Dissolved Organic Matter Isolates. *Environmental Science & Technology* **2007,** *41* (13), 4640-4646.
4. Fennell, B. D.; Mezyk, S. P.; McKay, G., Critical Review of UV-Advanced Reduction Processes for the Treatment of Chemical Contaminants in Water. *ACS Environmental Au* **2022**.
5. McKay, G.; Kleinman, J. L.; Johnston, K. M.; Dong, M. M.; Rosario-Ortiz, F. L.; Mezyk, S. P., Kinetics of the Reaction Between the Hydroxyl Radical and Organic Matter Standards from the International Humic Substance Society. *Journal of Soils and Sediments* **2013,** *14* (2), 298-304.
6. Lei, X.; Lei, Y.; Guan, J.; Westerhoff, P.; Yang, X., Kinetics and Transformations of Diverse Dissolved Organic Matter Fractions with Sulfate Radicals. *Environmental Science & Technology* **2022,** *56* (7), 4457-4466.
7. Lei, Y.; Lei, X.; Westerhoff, P.; Zhang, X.; Yang, X., Reactivity of Chlorine Radicals ($Cl^{\bullet}$ and $Cl_2^{\bullet -}$) with Dissolved Organic Matter and the Formation of Chlorinated Byproducts. *Environmental Science & Technology* **2021,** *55* (1), 689-699.
8. Yan, S.; Liu, Y.; Lian, L.; Li, R.; Ma, J.; Zhou, H.; Song, W., Photochemical Formation of Carbonate Radical and its Reaction with Dissolved Organic Matters. *Water Res* **2019,** *161*, 288-296.
9. Acidic Functional Groups of IHSS Samples. https://humic-substances.org/acidic-functional-groups-of-ihss-samples/ (accessed March 30, 2022).
10. Aeschbacher, M.; Graf, C.; Schwarzenbach, R. P.; Sander, M., Antioxidant Properties of Humic Substances. *Environmental Science & Technology* **2012,** *46* (9), 4916-25.
11. Li, H.; McKay, G., Relationships between the Physicochemical Properties of Dissolved Organic Matter and Its Reaction with Sodium Borohydride. *Environ Sci Technol* **2021,** *55* (15), 10843-10851.
12. Elemental Compositions and Stable Isotopic Ratios of IHSS Samples. https://humic-substances.org/elemental-compositions-and-stable-isotopic-ratios-of-ihss-samples/ (accessed March 30, 2022).
13. ^{13}C NMR Estimates of Carbon Distribution. https://humic-substances.org/13c-nmr-estimates-of-carbon-distribution-in-ihss-samples/ (accessed March 30, 2022).
14. Li, X.; Ma, J.; Liu, G.; Fang, J.; Yue, S.; Guan, Y.; Chen, L.; Liu, X., Efficient Reductive Dechlorination of Monochloroacetic Acid by Sulfite/UV Process. *Environ Sci Technol* **2012,** *46* (13), 7342-9.
15. Fennell, B. D.; Odorisio, A.; McKay, G., Quantifying Hydrated Electron Transformation Kinetics in UV-Advanced Reduction Processes Using the $R_{e-,UV}$ Method. *Environmental Science & Technology* **2022,** *56* (14), 10329-10338.
